# A Bottom-Up Whole-Body Physiologically Based Pharmacokinetic Model to Mechanistically Predict Tissue Distribution and the Rate of Subcutaneous Absorption of Therapeutic Proteins

**DOI:** 10.1208/s12248-015-9819-4

**Published:** 2015-09-25

**Authors:** Katherine L. Gill, Iain Gardner, Linzhong Li, Masoud Jamei

**Affiliations:** Simcyp (A Certara Company), Blades Enterprise Centre, John Street, Sheffield, S2 4SU UK

**Keywords:** PBPK, pharmacokinetics, simulation, subcutaneous absorption, therapeutic protein

## Abstract

The ability to predict subcutaneous (SC) absorption rate and tissue distribution of therapeutic proteins (TPs) using a bottom-up approach is highly desirable early in the drug development process prior to clinical data being available. A whole-body physiologically based pharmacokinetic (PBPK) model, requiring only a few drug parameters, to predict plasma and interstitial fluid concentrations of TPs in humans after intravenous and subcutaneous dosing has been developed. Movement of TPs between vascular and interstitial spaces was described by considering both convection and diffusion processes using a 2-pore framework. The model was optimised using a variety of literature sources, such as tissue lymph/plasma concentration ratios in humans and animals, information on the percentage of dose absorbed following SC dosing *via* lymph in animals and data showing loss of radiolabelled IgG from the SC dosing site in humans. The resultant model was used to predict *t*_max_ and plasma concentration profiles for 12 TPs (molecular weight 8–150 kDa) following SC dosing. The predicted plasma concentration profiles were generally comparable to observed data. *t*_max_ was predicted within 3-fold of reported values, with one third of the predictions within 0.8–1.25-fold. There was no systematic bias in simulated *C*_max_ values, although a general trend for underprediction of *t*_max_ was observed. No clear trend between prediction accuracy of *t*_max_ and TP isoelectric point or molecular size was apparent. The mechanistic whole-body PBPK model described here can be applied to predict absorption rate of TPs into blood and movement into target tissues following SC dosing.

**Electronic supplementary material:**

The online version of this article (doi:10.1208/s12248-015-9819-4) contains supplementary material, which is available to authorized users.

## INTRODUCTION

Therapeutic proteins (TPs) have been used clinically for many years (e.g. insulin, erythropoietin (EPO), growth hormone), and with the more recent development of monoclonal antibodies (mAbs), fusion proteins, antibody-drug conjugates, etc. represent a fast-growing sector of pharmaceutical development (1,2). Subcutaneous (SC) dosing is a common administration route for TPs, which cannot usually be given orally due to their poor bioavailability (3,4).

SC dosing delivers drugs into the interstitial space of the hypodermis, located between the skin and the muscle. The thickness and structure of the hypodermis varies between species and also with body location (5). The interstitial space is the area between the capillary endothelial cells and the tissue cells themselves (6). There have been several reviews of the structure of the interstitial space and the transport of proteins from the interstitium into the blood and lymph (5–9); therefore, only brief details will be given here. The interstitium is filled with extracellular matrix, comprised mainly of collagen, elastin and glycosaminoglycans. Together these elements give the interstitial fluid a gel-like consistency and a net negative charge, which influences drug distribution and transport at the administration site (5). From the interstitial space, drugs can gain access to the systemic circulation by either direct diffusion/transport across the endothelial cells into capillaries or by movement with the convective flow of interstitial fluid into the lymphatic vessels, which eventually drain into the blood.

Due to their size and polarity, TPs have limited direct diffusion across endothelial cell membranes and movement to the blood occurs mainly *via* diffusion and convection through pores in the endothelial wall, which is limited by protein size (6,7,10). For large TPs, a substantial portion of absorption into the systemic circulation following SC administration occurs *via* the lymphatic system (11–14). Supersaxo *et al.* (13) showed a positive correlation between increasing protein size and the contribution of lymphatic absorption following SC dosing in sheep (11–14). As lymph flow is much slower than blood flow from the tissues (7), absorption *via* the lymphatics is likely to contribute to the late maximum concentration (*C*_max_) observed following SC administration of many TPs (7,12,14).

Several pharmacokinetic (PK) models have been constructed to describe/predict the rate and extent of SC absorption of TPs; these have been reviewed recently (15). The vast majority of these models are empirical in nature and require fitting of clinical data to parameterise the models, hindering the prediction of SC absorption in early drug development when such data are unavailable. In addition, the accuracy of the prediction of SC absorption and bioavailability using allometry of animal data is inadequate (5). Ibrahim *et al.* (11) presented a PK model for dermal clearance, where lymph and blood absorption of free and protein-bound solutes was described based on the 2-pore hypothesis. The model predicted blood capillary permeability and percentage of dose absorbed through the lymph for a variety of solutes with good accuracy and precision relative to the observed clinical data (11). However, this model was not linked to a PK model describing drug disposition in the rest of the body. Therefore, the model predictions for absorption could not be compared to clinical data for *C*_max_ and time of *C*_max_ (*t*_max_). In addition, the dermal clearance model could not account for the return of drug to the interstitial fluid at the SC site *via* recirculation which is known to be an important factor in interpretation of experimental data (15). A whole-body physiologically based PK (PBPK) model incorporating the SC dosing site as part of the skin was reported recently (16). This model accounted for the recirculation of TP to the SC site and allowed prediction of *C*_max_ and *t*_max_. However, the movement of protein was based solely on lymphatic transport and hence the model may not be suitable for smaller TPs where direct absorption of drug into blood at the SC site may be an important absorption route (13).

In the current study, a whole-body PBPK model has been developed to mechanistically predict the rate of SC absorption and plasma and interstitial fluid concentrations of TPs in humans. The model requires a limited number of drug parameters which makes it suitable even at the early stage of drug development. The model predicts the TP absorption rate and tissue distribution based upon the molecular size of the protein using a 2-pore framework (10,17,18). A limitation of the model is that at the moment, bioavailability cannot be predicted mechanistically from *in vitro* data, so an empirical estimate of bioavailability is needed. The prediction accuracy of tissue distribution at steady state, plasma concentration profiles and *t*_max_ following SC dosing of TPs, including both small TPs and mAbs, using the PBPK model is presented.

## MATERIALS AND METHODS

### Structure of the PBPK Model

**Fig. 1:**
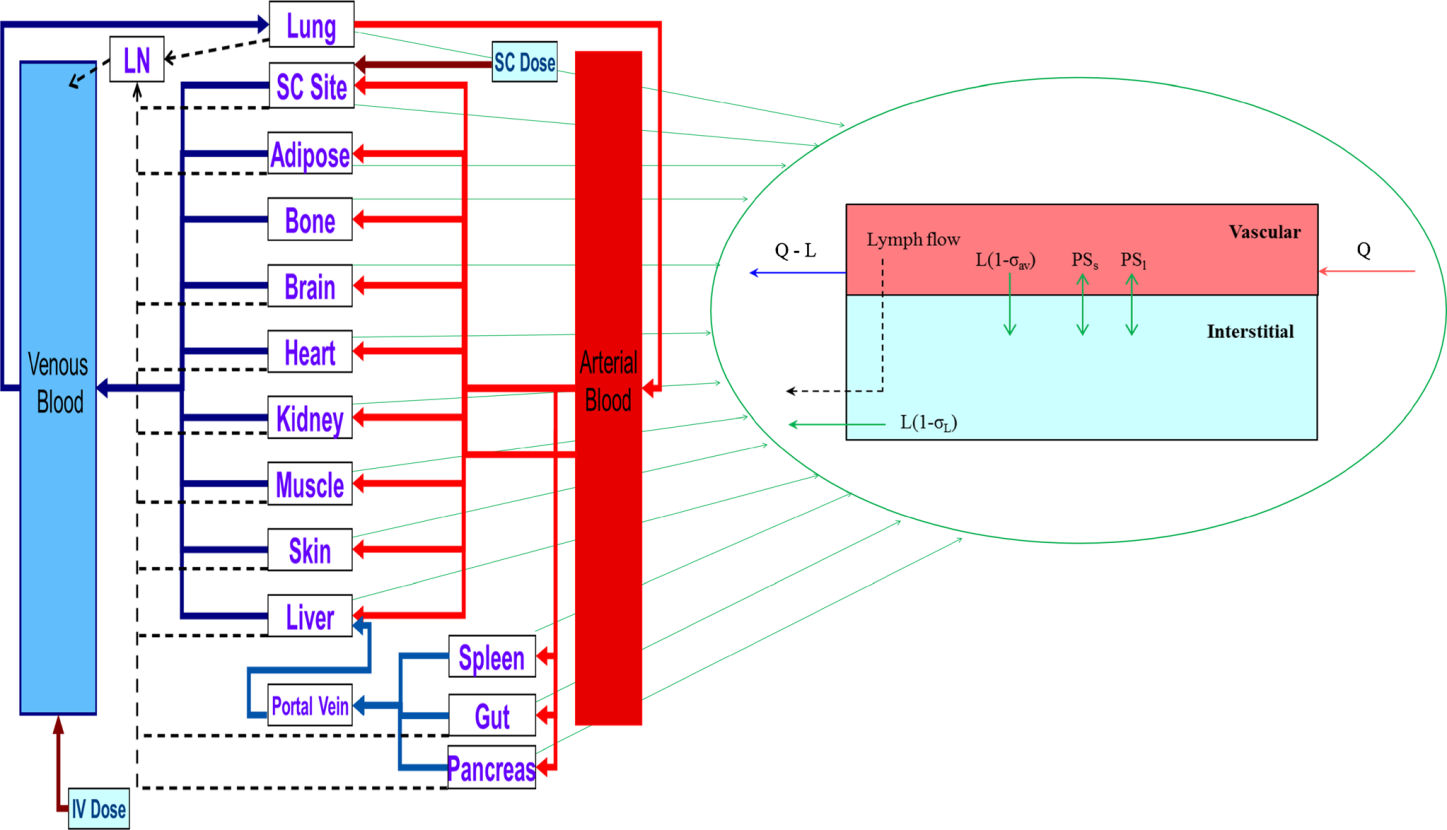
Structure of the permeability limited tissue model incorporated into the whole-body PBPK model for therapeutic proteins. *Solid red* and *blue lines* represent arterial and venous blood flow; *dashed black lines* represent lymph flow. *LN*, *L*, *Q*, *PS* and *σ* represent central lymph, lymph flow, blood flow, permeability surface area product and reflection coefficient, respectively

A human whole-body PBPK model was developed and implemented in the Simcyp Simulator (V14 R1, Simcyp, Sheffield, UK). The model contains 11 tissues, each being described by two compartments, representing vascular and interstitial spaces (Fig. 1). This tissue structure was also used to represent the SC dosing site. In addition to the flow of blood to and from each organ, the flow of lymph from individual tissues is accounted for. The lymph flow from each tissue in the PBPK model is collected into a single compartment (central lymph), and from here, the total lymph flow is returned to the venous circulation, maintaining fluid balance (Fig. 1). The differential equations used to describe the movement of TP in the PBPK model are shown below (Eqs. 1 to 5).

1 $$ V{\mathrm{v}}_{\mathrm{org}}\times \frac{d{C}_{\mathrm{v},\mathrm{o}\mathrm{r}\mathrm{g}}}{dt}=\left({Q}_{\mathrm{org}}\times {C}_{\mathrm{ab}}\right)-\left({Q}_{\mathrm{org}}-{L}_{\mathrm{org}}\right)\times {C}_{\mathrm{v},\mathrm{o}\mathrm{r}\mathrm{g}} - {L}_{\mathrm{org}}\times \left(1-{\sigma}_{\mathrm{av},\mathrm{o}\mathrm{r}\mathrm{g}}\right)\times {C}_{\mathrm{v},\mathrm{o}\mathrm{r}\mathrm{g}}-\left(P{S}_{\mathrm{s},\mathrm{o}\mathrm{r}\mathrm{g}}\times \frac{P{e}_{\mathrm{s},\mathrm{o}\mathrm{r}\mathrm{g}}}{e^{P{e}_{\mathrm{s},\mathrm{o}\mathrm{r}\mathrm{g}}}-1}+P{S}_{\mathrm{l},\mathrm{o}\mathrm{r}\mathrm{g}}\times \frac{P{e}_{\mathrm{l},\mathrm{o}\mathrm{r}\mathrm{g}}}{e^{P{e}_{\mathrm{l},\mathrm{o}\mathrm{r}\mathrm{g}}}-1}\right)\times \left({C}_{\mathrm{v},\mathrm{o}\mathrm{r}\mathrm{g}}-{C}_{\mathrm{i},\mathrm{o}\mathrm{r}\mathrm{g}}\right) $$

where the subscript org indicates the organ (adipose, bone, brain, gut, heart, lung, muscle, pancreas, skin, spleen and SC site) and *V*v_org_, *C*_v,org_, *Q*_org_, *C*_ab_, *L*_org_, *σ*_av,org_, *PS*_s,org_, *PS*_l,org_, *Pe*_s,org_, *Pe*_l,org_ and *C*_i,org_ are the vascular space volume, vascular space concentration, blood flow, concentration in arterial blood, lymph flow, average vascular reflection coefficient, permeability surface area product (PS) through small pores, PS through large pores, small pore peclet number, large pore peclet number and interstitial fluid concentration, respectively. For the lung, *Q*_org_ represents the entire cardiac output. *σ*_av,org_ takes into account the fractional total hydraulic conductance accounted for by small and large pores and the osmotic reflection coefficient for small and large pores in a given organ (10).

2 $$ {V}_{\mathrm{i},\mathrm{o}\mathrm{r}\mathrm{g}}\times \frac{d{C}_{\mathrm{i},\mathrm{o}\mathrm{r}\mathrm{g}}}{dt} = {L}_{\mathrm{org}}\times \left(1-{\sigma}_{\mathrm{av},\mathrm{o}\mathrm{r}\mathrm{g}}\right)\times {C}_{\mathrm{v},\mathrm{o}\mathrm{r}\mathrm{g}}+\left(P{S}_{\mathrm{s},\mathrm{o}\mathrm{r}\mathrm{g}}\times \frac{P{e}_{\mathrm{s},\mathrm{o}\mathrm{r}\mathrm{g}}}{e^{P{e}_{\mathrm{s},\mathrm{o}\mathrm{r}\mathrm{g}}}-1}+P{S}_{\mathrm{l},\mathrm{o}\mathrm{r}\mathrm{g}}\times \frac{P{e}_{\mathrm{l},\mathrm{o}\mathrm{r}\mathrm{g}}}{e^{P{e}_{\mathrm{l},\mathrm{o}\mathrm{r}\mathrm{g}}}-1}\right)\times \left({C}_{\mathrm{v},\mathrm{o}\mathrm{r}\mathrm{g}}-{C}_{\mathrm{i},\mathrm{o}\mathrm{r}\mathrm{g}}\right) - {L}_{\mathrm{org}}\times \left(1-{\sigma}_{L,\mathrm{o}\mathrm{r}\mathrm{g}}\right)\times {C}_{\mathrm{i},\ \mathrm{o}\mathrm{r}\mathrm{g}} $$

where *V*_i,org_ and *σ*_L,org_ are the interstitial space volume and lymph reflection coefficient, respectively.

3 $$ {V}_{\mathrm{LN}}\times \frac{d{C}_{\mathrm{LN}}}{dt} = {\displaystyle \sum_{\mathrm{tissues}}\left({L}_{\mathrm{org}}\times \left(1-{\sigma}_{L,\mathrm{o}\mathrm{r}\mathrm{g}}\right)\times {C}_{\mathrm{i},\mathrm{o}\mathrm{r}\mathrm{g}}\right) - {L}_{\mathrm{total}}\times {C}_{\mathrm{LN}}} $$

where *V*_LN_, *C*_LN_ and *L*_total_ are the central lymph compartment volume, the central lymph compartment concentration and total lymph flow (the sum of *L*_org_ for all tissues), respectively. The summation here is for all tissues.

4 $$ {V}_{\mathrm{v}\mathrm{b}}\times \frac{d{C}_{\mathrm{v}\mathrm{b}}}{dt}=\left({\displaystyle \sum_{\mathrm{tissues}}\left({Q}_{\mathrm{org}}-{L}_{\mathrm{org}}\right)\times {C}_{\mathrm{v},\mathrm{o}\mathrm{r}\mathrm{g}}}\right)-{Q}_{\mathrm{C}}\times {C}_{\mathrm{v}\mathrm{b}}+{L}_{\mathrm{total}}\times {C}_{\mathrm{LN}} $$

where *V*_vb_, *C*_vb_ and *Q*_c_ are the venous blood volume, concentration in venous blood and cardiac output, respectively. The summation here is for all tissues except lung, spleen, gut and pancreas.

5 $$ {V}_{\mathrm{ab}}\times \frac{d{C}_{\mathrm{ab}}}{dt}=\left({Q}_{\mathrm{c}}-{L}_{\mathrm{lung}}\right)\times {C}_{\mathrm{v},\mathrm{lung}}-\left({Q}_{\mathrm{c}}-{L}_{\mathrm{lung}}\right)\times {C}_{\mathrm{ab}} - \frac{C{L}_{\mathrm{p}}}{BP}\times {C}_{\mathrm{ab}} $$

where *V*_ab_, *L*_lung_, *C*_v,lung_, *BP* and *CL*_p_ are the arterial blood volume, lung lymph flow, lung vascular space concentration, blood/plasma concentration ratio and plasma clearance, respectively. Here, flow balance has been imposed, i.e*.* the flow into the arterial blood equals to the flow out of this compartment.

Some alterations to Eq. 1 were required for the liver vascular space, as detailed in Eq. 6.

6 $$ \begin{array}{l}V{\mathrm{v}}_{\mathrm{l}\mathrm{iver}}\times \frac{d{C}_{\mathrm{v},\mathrm{liver}}}{dt}=\left({Q}_{\mathrm{l}\mathrm{iver}}\times {C}_{\mathrm{ab}}\right)+\left({Q}_{\mathrm{gut}}-{L}_{\mathrm{gut}}\right)\times {C}_{\mathrm{v},\mathrm{gut}} + \left({Q}_{\mathrm{s}\mathrm{pleen}}-{L}_{\mathrm{s}\mathrm{pleen}}\right)\times {C}_{\mathrm{v},\mathrm{spleen}} + \left({Q}_{\mathrm{pancrea}s}-{L}_{\mathrm{pancrea}\mathrm{s}}\right)\times {C}_{\mathrm{v},\mathrm{pancreas}}-\left(\left({Q}_{\mathrm{gut}}-{L}_{\mathrm{gut}}\right)+\left({Q}_{\mathrm{s}\mathrm{pleen}}-{L}_{\mathrm{s}\mathrm{pleen}}\right)+\left({Q}_{\mathrm{pancrea}\mathrm{s}}-{L}_{\mathrm{pancrea}\mathrm{s}}\right)+\left({Q}_{\mathrm{l}\mathrm{iver}}-{L}_{\mathrm{l}\mathrm{iver}}\right)\right)\times {C}_{\mathrm{v},\mathrm{liver}}\\ {} - {L}_{\mathrm{l}\mathrm{iver}}\times \left(1-{\sigma}_{\mathrm{av},\mathrm{liver}}\right)\times {C}_{\mathrm{v},\mathrm{liver}}-\left(P{S}_{\mathrm{s},\mathrm{liver}}\times \frac{P{e}_{\mathrm{s},\mathrm{liver}}}{e^{P{e}_{\mathrm{s},\mathrm{liver}}}-1}+P{S}_{\mathrm{l},\mathrm{liver}}\times \frac{P{e}_{\mathrm{l},\mathrm{liver}}}{e^{P{e}_{\mathrm{l},\mathrm{liver}}}-1}\right)\times \left({C}_{\mathrm{v},\mathrm{liver}}-{C}_{\mathrm{i},\mathrm{liver}}\right)\end{array} $$

where *V*v_liver_, *C*_v,liver_, *Q*_liver_, *Q*_gut_, *L*_gut_, *C*_v,gut_, *Q*_spleen_, *L*_spleen_, *C*_v,spleen_, *Q*_pancreas_, *L*_pancreas_, *C*_v,pancreas_, *L*_liver_, *σ*_av,liver_, *PS*_s,liver_, *Pe*_s,liver_, *PS*_l,liver_, *Pe*_l,liver_ and *C*_i,liver_, are the liver vascular space volume, liver vascular space concentration, hepatic artery blood flow, gut blood flow, gut lymph flow, gut vascular space concentration, spleen blood flow, spleen lymph flow, spleen vascular space concentration, pancreas blood flow, pancreas lymph flow, pancreas vascular space concentration, liver lymph flow, liver average vascular reflection coefficient, liver PS through small pores, liver small pore peclet number, liver PS through large pores, liver large pore peclet number and liver interstitial fluid concentration, respectively. Q_liver_ represents 19% of cardiac output (19).

SC dose was described as a bolus input to the interstitial compartment of the SC dosing site. The initial concentration for the SC interstitial space is defined as (dose × *F*)/*V*_int,SC site_, where *F* is the bioavailability. For all the other compartments in the PBPK model, the initial concentration is 0.

### System Parameters

System parameters were taken from a population representative Sim-Healthy Volunteer simulation in the Simcyp Simulator V14R1. Values for whole organ volume, fraction of vascular space, fraction of extracellular water and blood flow to each tissue are given in Table I; these parameters are the same as those used for modelling of small molecule drugs in Simcyp (20,21). The body weight and cardiac output were 80.7 kg and 356 L/h, respectively. The remaining blood flow, lymph flow and body volume were assigned to a ‘bypass’ compartment to ensure mass balance. The interstitial space, venous blood and arterial blood volumes are calculated from Eqs. 7 to 9.

**Table I:** System Parameters used in the Whole-Body PBPK Model for Describing the Pharmacokinetics of Therapeutic Proteins

Tissue	Whole organ volume (L)	Fraction of vascular space	Fraction of extracellular water	% cardiac output	% total lymph flow	Small pore radius (nm)	Large pore radius (nm)	Small pore/large pore
Adipose	22.7	0.031	0.141	5.00	12.8	7.0^*b*^	20^*a*^	500^*a*^
Bone	3.95	0.05	0.098	5.00	0.00	9.0^*a*^	33^*b*^	46^*a*^
Brain	1.34	0.05	0.092	12.0	1.05	0.6^*b*^	18	20,000,000^*b*^
Gut	1.22	0.05	0.267	15.0	12.0	4.8	25^*b*^	500^*a*^
Heart	0.359	0.042	0.313	4.00	1.00	4.8	25	400^*b*^
Kidney	0.325	0.07	0.283	19.0	8.50	7.4	20	200^*b*^
Liver	1.61	0.05	0.165	25.5	33.0	9.0	33	46
Lung	0.547	0.185	0.348	100	3.00	9.0^*b*^	25^*b*^	45^*b*^
Muscle	31.3	0.027	0.091	17.0	16.0	4.5	22	2000^*b*^
Pancreas	0.123	0.05	0.12	0.0100	0.30	6.0^*a*^	20^*a*^	3610^*a*^
Skin	3.15	0.05	0.623	5.00	7.30	6.0^*a*^	20^*a*^	500^*a*^
Spleen	0.150	0.05	0.208	0.0200	0.00	9.0^*a*^	33^*a*^	46^*a*^
SC site	0.005	0.05	0.623	0.0160	0.0392	5.0^*a*^	20^*a*^	500^*a*^
Arterial blood	1.16							
Venous blood	2.33							
Central lymph	0.312							

System parameters based on the Population Representative Sim-Healthy Volunteer in the Simcyp Simulator V14R1. Whole organ volume, fraction of vascular space, fraction of extracellular water and blood flow to each tissue (20,21); full references for lymph flow, pore sizes and large pore/small pore values (prior to optimisation) can be found in the Supplemental Material

^*a*^No observed data available, values optimised to recover observed lymph/plasma concentration data

^*b*^Observed values optimised to recover observed lymph/plasma concentration data

7 $$ {V}_{\mathrm{i},\mathrm{o}\mathrm{r}\mathrm{g}} = \left(\mathrm{total}\ \mathrm{organ}\ \mathrm{volume} \times {F}_{\mathrm{EW}}\right)\ \hbox{--}\ V{\mathrm{v}}_{\mathrm{org}} $$

where *F*_EW_ is the fraction of extracellular water in the tissue.

8 $$ {V}_{\mathrm{v}\mathrm{b}}=\left(\mathrm{total}\ \mathrm{b}\mathrm{lood}\ \mathrm{volume}-{\displaystyle {\sum}_{\mathrm{tissues}}\kern0.5em }V{\mathrm{v}}_{\mathrm{org}}\right)\times \frac{2}{3} $$

9 $$ {V}_{\mathrm{ab}}=\left(\mathrm{total}\ \mathrm{blood}\ \mathrm{volume} - {\displaystyle {\sum}_{\mathrm{tissues}}\kern0.5em }V{\mathrm{v}}_{\mathrm{org}}\right)\times \frac{1}{3} $$

Lymph flow to each tissue and total lymph flow data for humans were collated from the literature where possible. Due to reabsorption of fluid in the lymph nodes, the lymph flow measured in the thoracic duct or other sites that are distal to the lymph nodes may give a lower value of fluid flow than that which drains from the interstitial spaces of the tissues (6). In the PBPK model, it was assumed that lymph node fluid reabsorption is negligible, and therefore, the estimate of total lymph flow (0.00386 L/h/kg) reflects the summation of the flow of fluid from the blood into the interstitial space of all of the tissues combined. The percentage of total lymph flow returning from each individual tissue is detailed in Table I. Estimates were based on data collated from the literature for humans or allometrically scaled from animals. Where a range of values were found, a weighted mean value was chosen. The spleen and bone do not have lymph vessels exiting the tissue, and so lymph flow was set to 0 L/h (6,22–25).

The time course of protein in spleen and bone was modelled using parameters that ensure rapid equilibration between the vascular and interstitial spaces (*PS*_s,org_ and *PS*_l,org_ = 0.1, and *Pe*_s,org_/*e* ^*Pes*,org^ − 1 and *Pe*_l,org_/*e* ^*Pel*,org^ − 1 = 1) and hence operate similar to well stirred compartments.

Movement of TPs between the vascular and interstitial spaces is described mechanistically by considering convection and diffusion processes using a 2-pore model (6,10). This model assumes that the endothelial membrane contains pores allowing the flow of fluid and proteins between the vascular and interstitial spaces. The pores in the endothelial membrane are considered to be of two discrete sizes; large and small pores. For each tissue, the pore sizes and the relative frequency of the large and small pores were defined by collation of data from the literature where available and manual optimisation when the values were not available (see the Model Validation section). Optimisation was performed by fixing the tissue volumes and blood and lymph flows and manually adjusting the pore sizes and relative frequency of the large and small pores by trial and error until the predicted concentration ratio of protein in plasma, and the lymph was comparable to observed data. The pore radii and the ratio of small pores to large pores in each tissue are given in Table I.

### Drug-Specific Parameters

The assumptions and derivation of the 2-pore model have been detailed extensively in previous publications, and interested readers are referred to the following references (6,10). Briefly, this model describes the convection and diffusion of proteins through the pores in the endothelial membrane based on the radius of the pore relative to the hydrodynamic radius (Rs) of the TP. If the TP is large compared to the pore (Rs > radius of the pore), then there will be no movement of the TP through that particular set of pores. The methods used to calculate values of *σ*_av_, *PS*_s_, *PS*_l_, *Pe*_s_ and *Pe*_l_ in each of the tissues are detailed in references (6,10). The Rs of each TP was calculated from molecular weight using Simcyp V14 R1. The movement of TP from interstitial space into lymph is not considered to be restrictive and therefore *σ*_L_ is set to 0 for all tissues and TPs. Binding is not considered within the lymphatics of the PBPK model.

### Model Validation

#### Full PBPK Model

Plasma (Cp) and tissue Ci concentrations at steady state were simulated for theoretical TPs covering a range of Rs (1–11 nm). CL_p_ was set to zero to ensure steady-state concentrations were achieved in all compartments of the PBPK model during the simulations. The simulated Ci/Cp ratios were compared with literature values of lymph/plasma concentration ratios from a variety of proteins for tissues in humans and experimental animals, with the assumption that lymph concentrations exiting tissues are a measurable surrogate of Ci at steady state (26) (references for the collated literature values are given in the Supplemental Material).

#### Development of the SC Site Model

Physiological parameters for a 5 mL volume were used to model the SC dosing site. The interstitial volume for the dosing site in the model was 3 mL, estimated using data for the diameter of the SC depot of radiolabelled IgG or albumin in skin with the assumption that the dose is confined to the interstitial fluid immediately following injection (Table II) (27–30).

**Table II:** Calculation of Lymph Flow and Interstitial Volume at the SC Site from Observed Radiolabelled IgG and Albumin Data Following SC Dosing

Protein	Number of subjects	Diameter (cm)	Radius (cm)	Volume (mL)^*a*^	K (%/min)	Lymph flow (mL/min)	Dosing site	Reference
Albumin	15	2.2	1.1	5.58^*b*^	NR	NR	Arm	(27)
IgG	8	1.41	0.71	1.47	0.157	0.00230	Hand	(28)
IgG	14	1.70	0.85	2.57	0.093	0.00239	Forearm	(29)
IgG	10	1.60	0.80	2.14	0.095	0.00204	Hand	(29)
		Weighted mean values		3.25	0.110	0.00226		

*NR* not reported, *K* drainage rate constant of IgG injected into SC tissue

^*a*^Volume calculated assuming IgG dose distributes into a spherical volume

^*b*^Calculated from a diffusion area of 3.8 cm^2^, assuming the area was for a circle

Observed data for the rate of radiolabelled IgG (28–30) loss from the SC dosing site were used to determine the lymph flow needed for the SC site. The lymph flow exiting the SC site was calculated under the assumption that IgG is too large to diffuse through endothelial pores; hence, all loss from the SC site is *via* lymph drainage, and there is no restriction to IgG entering the lymphatic system. Transcytosis of IgG *via* binding to the neonatal Fc receptor (FcRn) in the endothelial cells was not considered as it provides a minimal route of absorption (14,31), see Discussion section for more details. Lymph flow was calculated for each individual study using Eq. 10 (28–30) and the reported rate (K) and SC depot volume data calculated previously (Table II). The average fractional rate of loss for IgG was 0.0009725 min^−1^, providing an average SC site lymph flow of 0.00225 mL/min.

10 $$ K\ \mathrm{f}\mathrm{o}\mathrm{r}\ \mathrm{I}\mathrm{g}\mathrm{G}\ \mathrm{loss}\ \mathrm{f}\mathrm{r}\mathrm{o}\mathrm{m}\ \mathrm{S}\mathrm{C}\ \mathrm{site}=\frac{\mathrm{SC}\ \mathrm{site}\ \mathrm{lymph}\ \mathrm{f}\mathrm{low}}{\mathrm{Volume}\ \mathrm{o}\mathrm{f}\ \mathrm{S}\mathrm{C}\ \mathrm{depot}} $$

Cp and Ci concentrations at steady state were simulated for theoretical TPs with Rs of 1–11 nm and compared with literature values of lymph/plasma ratios for the SC site in humans and experimental animals to ensure that use of the pore radii and ratio of small/large pores for the skin were also suitable for the SC site. CL_p_ was set to 0 for the theoretical TPs. The model was optimised using percentage of dose absorbed in the lymph data reported for sheep (13). Unfortunately, such data from humans are lacking in the literature. Data from sheep were considered to give a more representative description of the percentage of dose absorbed in the lymph than data reported for scruff species such as rats and mice. This is because the structure of the SC tissue is markedly different in scruff species compared to higher mammals (5). Final model parameters are shown in Table I.

### Model Application

The model was then used to predict *t*_max_ and plasma concentration profiles for 12 TPs (MW 8–150 kDa) following SC dosing. The input parameters for each simulation are given in Table I of the Supplemental Material. Observed bioavailability and intravenous clearance values for each TP were collated from the literature. Where intravenous clearance data were unavailable, the values were determined using the parameter estimation facility in the Simcyp Simulator. The simulation results were compared with observed data from the literature. The observed concentration data were digitised using GetData graph digitiser version 2.22 (GetData Graph Digitizer, 2012, http://getdata-graph-digitizer.com/). Prediction accuracy for *t*_max_ was assessed using a measure of fold error. In addition, simulated *C*_max_ values were compared to observed values using the same method. Correlations between prediction accuracy of *C*_max_ or *t*_max_ and TP size were assessed. In addition, the relationship between prediction accuracy of *C*_max_ or *t*_max_ and TP isoelectric point (pI) was investigated.

### Sensitivity Analysis

Manual sensitivity analysis was performed to assess the impact of lymph flow on the *t*_max_ in the interstitial space and the steady-state Ci/Cp ratios. Hypothetical proteins with Rs of 1–7 nm were simulated with CL_p_ set to 0 L/h and with the dose administered as a bolus into the venous blood compartment. The total lymph flow was varied between 0.1- and 10-fold of the standard value.

## RESULTS

### Model Validation

**Fig. 2:**
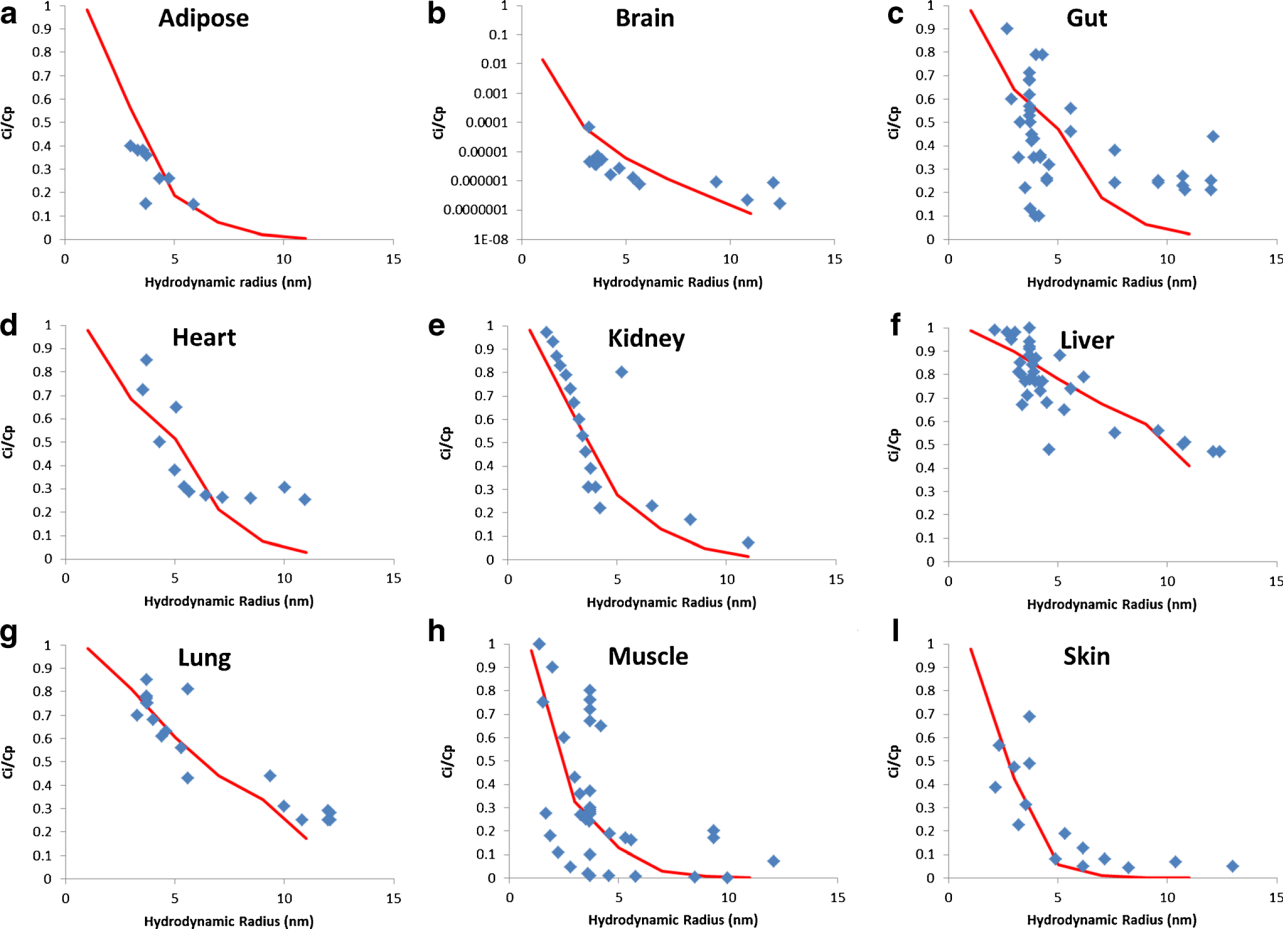
Predicted and observed Ci/Cp ratios for proteins with a range of hydrodynamic radii. **a** Adipose, **b** brain, **c** gut, **d** heart, **e** kidney, **f** liver, **g** lung, **h** muscle and **i** skin. *Blue diamonds* indicate observed data [References in Supplemental Material]; *Red line* denotes predicted data

Predicted and observed Ci/Cp ratios for each tissue are shown in Fig. 2; the bone, pancreas and spleen are not presented due to a lack of observed data in the literature. No obvious or systematic differences in observed Ci/Cp ratios were noted when data in experimental animals and humans (where available) were compared, so the entire experimental data set is presented. The predicted Ci/Cp ratios were similar to the observed data, showing that the model predicted protein distribution into the interstitial space well. For example, for a TP with radius of 3.55 nm (equivalent to albumin), the predicted Ci/Cp ratio was 0.87 for the liver, compared to Ci/Cp ratios of 0.78–1.00 reported *in vivo* (26).

### Development of the SC Site Model

**Fig. 3:**
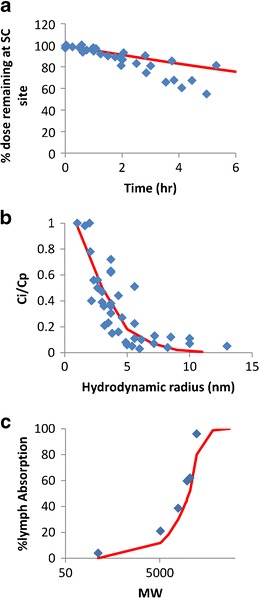
**a** Predicted and observed percentage of radiolabelled IgG dose remaining at the dosing site following bolus SC dosing; *Red line* denotes predicted data; *Blue diamonds* indicate observed data (28–30). **b** Predicted and observed Ci/Cp ratios for the SC site; *Red line* denotes predicted data; *Blue diamonds* indicate observed data (26,32–34). **c** Predicted and observed percentage of dose absorbed through the lymph for proteins of varying sizes; *Red line* denotes predicted data; *Blue diamonds* indicate observed data from sheep (13,35,36)

Observed data for the percentage of radiolabelled IgG dose remaining at the SC injection site over time (28–30) were plotted against the simulated data for a TP with hydrodynamic radius of 5 nm (Fig. 3a). The predicted Ci/Cp ratios for the SC site were comparable to the observed values collated from the literature (26,32–34), as shown in Fig. 3b. Therefore, the pore radii and ratio of small/large pores for the skin were suitable for the SC site. The predicted percentage of dose absorbed through the lymph for proteins with a range of sizes compared to values from sheep (13) are shown in Fig. 3c.

### Model Application

**Fig. 4:**
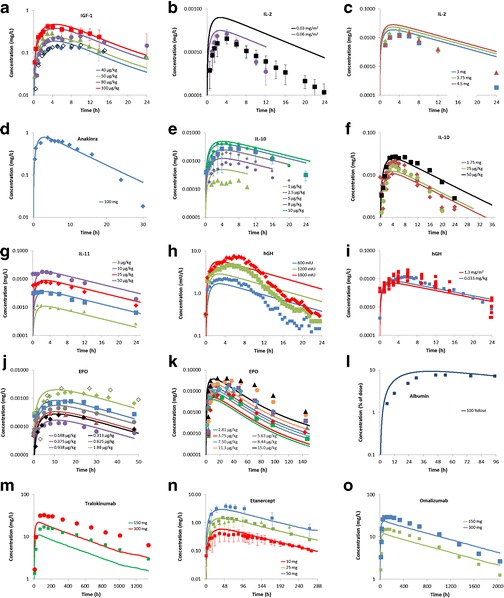
Predicted and observed plasma concentrations for TPs following SC dosing. **a** IGF-1; **b**, **c** IL-2; **d** Anakinra; **e, f** IL-10; **g** IL-11; **h**, **i** hGH; **j**, **k** EPO; **l** Albumin; **m** Tralokinumab; **n** Etanercept; **o** Omalizumab. *Symbols* represent observed data; *lines* represent predicted data. **a**: *blue*, *open diamond*, *green, purple and red symbols/lines* = 40, 40, 50, 80 and 100 μg/kg doses (37–39); **b**: *black and purple symbols/lines* = 0.03 and 0.06 mg/m^2^ doses (40); **c**: *blue, green and red symbols/lines* = 3, 3.75 and 4.5 mg doses (41); **d**: *blue symbols/line* = 100 mg dose (42); **e**: *olive green, purple, grey, blue and green symbols/lines* = 1, 2.5, 5, 8 and 10 μg/kg doses (43,45); **f**: *red, green and black symbols/lines* = 1.75 mg, 25 and 50 μg/kg doses (44,45); **g**: *green, blue, red and purple symbols/lines* = 3, 10, 25 and 50 μg/kg doses (46); **h**: *blue, green and red symbols/lines* = 600, 1200 and 1800 mIU doses (47); **i**: *red and blue symbols/lines* = 1.3 mg/m^2^ and 0.033 mg/kg doses (48,49); **j**: *purple, black, red, grey, blue, green and open diamond symbols/lines* = 0.188, 0.313, 0.375, 0.625, 0.938, 1.88 and 1.88 μg/kg doses (50–53); **k**: *green, red, grey, blue, purple, orange and black symbols/lines* = 2.81, 3.75, 5.63, 7.50, 8.44, 11.3 and 15 μg/kg doses (50); **l**: *blue symbols/line* = 100% of dose (27); **m**: *green and red symbols/lines* = 150 and 300 mg doses (54); **n**: *red, green and blue symbols/lines* = 10, 25 and 50 mg doses (55–58); **o**: *green and blue symbols/lines* = 150 and 300 mg doses (59)

**Fig. 5:**
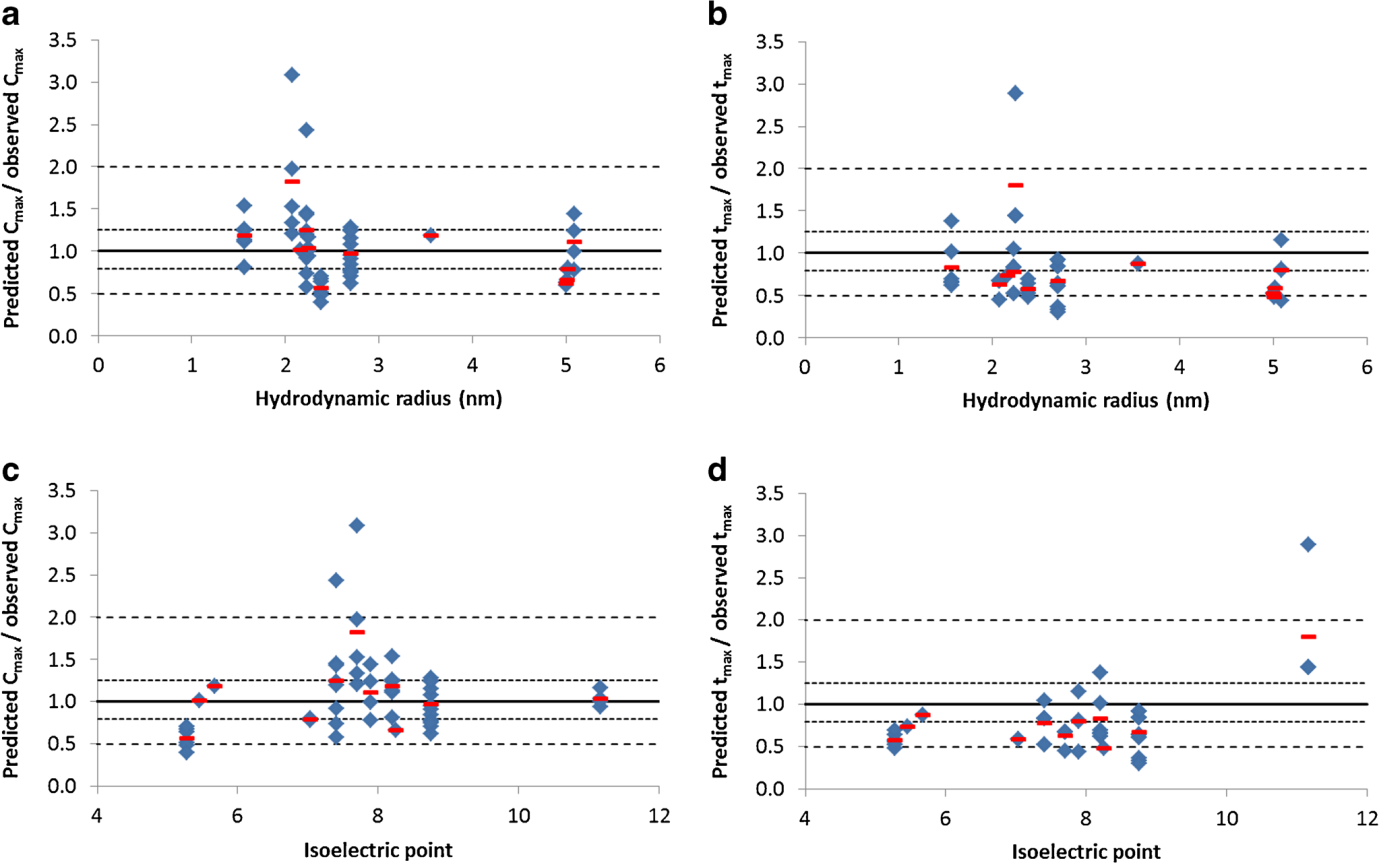
Prediction accuracy of *C*_max_ (**a**, **c**) and *t*_max_ (**b**, **d**) compared to hydrodynamic radius (**a**, **b**) or isoelectric point (**c**, **d**). *Blue diamonds* denote prediction accuracy for individual studies/dose levels; *Red line* represents mean prediction accuracy for each TP; *Black line* indicates line of unity; . . . 0.8 to 1.25-fold prediction accuracy; - - - 2-fold prediction accuracy

The dataset of observed concentration profiles following SC dosing contained 54 studies/dose levels, with up to 14 sets of observed data per TP. Simulated plasma concentration profiles following SC dosing for the included TPs were generally similar to observed data (Fig. 4, linear plots are shown in Supplemental Material Fig. 1). The prediction accuracy of *C*_max_ and *t*_max_ for the complete dataset and summary statistics are presented in Table III. Simulated *C*_max_ was within 3.1-fold of observed values, with approximately half (46%) of the simulated *C*_max_ values falling within 0.8–1.25-fold of the observed values. A third (31%) of *t*_max_ predictions were within 0.8–1.25-fold of observed values, with all predictions falling within 3.3-fold. There was no systematic bias for over or underprediction of *C*_max_, although a general trend for underprediction of *t*_max_ was apparent (Fig. 5). The extent of the *t*_max_ underprediction did not correlate with the molecular size of the TP (Fig. 5b). For TPs with molecular sizes < 150 kDa, *t*_max_ was generally predicted within 0.30- and 2.9-fold of observed values, similarly for mAbs (molecular weight ~150 kDa), the *t*_max_ was predicted within 0.44- and 1.2-fold of observed values (Fig. 5b). In addition, no clear trend between prediction accuracy of *C*_max_ or *t*_max_ and TP pI was apparent (Fig. 5c, d).

**Table III:** Prediction Accuracy of *C*_max_ and *t*_max_ Following Subcutaneous Dosing of Therapeutic Proteins with a Range of Sizes using the PBPK Model

Protein	Molecular weight (kDa)	Hydrodynamic radius (nm)	Isoelectric point	Dose (mg/kg)	Reference	Predicted		Predicted/observed	
						*C*_max_ (mg/L)	*t*_max_ (h)	*C*_max_	*t*_max_
IGF-1	7.6	1.56	8.2	0.040	(37)	0.188	4.59	1.11	0.62
IGF-1	7.6	1.56	8.2	0.080	(37)	0.377	4.58	1.24	0.69
IGF-1	7.6	1.56	8.2	0.050	(38)	0.235	4.59	0.82	1.38
IGF-1	7.6	1.56	8.2	0.100	(38)	0.471	4.58	1.13	1.02
IGF-1	7.6	1.56	8.2	0.040	(39)	0.188	4.59	1.26	0.67
IGF-1	7.6	1.56	8.2	0.080	(39)	0.377	4.58	1.54	0.65
IL-2	15.5	2.07	7.7	0.0292^*a*^	(40)	0.000316	2.70	1.21	0.68
IL-2	15.5	2.07	7.7	0.0583^*a*^	(40)	0.000632	2.70	3.08	0.67
IL-2	15.5	2.07	7.7	3.00^*b*^	(41)	0.0188	2.70	1.53	0.68
IL-2	15.5	2.07	7.7	3.75^*b*^	(41)	0.0235	2.70	1.33	0.67
IL-2	15.5	2.07	7.7	4.50^*b*^	(41)	0.0282	2.70	1.97	0.45
Anakinra	17.3	2.16	5.5	100^*c*^	(42)	0.782	2.93	1.01	0.73
IL-10	18.7	2.23	7.4	0.008	(43)	0.00397	4.18	1.46	0.52
IL-10	18.7	2.23	7.4	1.75^*c*^	(44)	0.0108	4.19	0.58	0.84
IL-10	18.7	2.23	7.4	0.001	(45)	0.000496	4.17	2.43	0.52
IL-10	18.7	2.23	7.4	0.0025	(45)	0.00124	4.18	1.43	0.84
IL-10	18.7	2.23	7.4	0.005	(45)	0.00248	4.19	1.23	0.84
IL-10	18.7	2.23	7.4	0.010	(45)	0.00496	4.18	1.19	0.84
IL-10	18.7	2.23	7.4	0.025	(45)	0.0124	4.18	0.74	1.04
IL-10	18.7	2.23	7.4	0.050	(45)	0.0248	4.19	0.92	0.84
IL-11	19	2.25	11.2	0.003	(46)	0.00108	2.89	0.94	2.89
IL-11	19	2.25	11.2	0.010	(46)	0.00361	2.89	0.94	1.44
IL-11	19	2.25	11.2	0.025	(46)	0.00902	2.89	1.17	1.44
IL-11	19	2.25	11.2	0.050	(46)	0.0180	2.88	1.01	1.44
hGH	22	2.38	5.27	600^*d*^	(47)	1.69^*d*^	2.77	0.64	0.65
hGH	22	2.38	5.27	1200^*d*^	(47)	2.87^*d*^	2.77	0.52	0.58
hGH	22	2.38	5.27	1800^*d*^	(47)	5.67^*d*^	2.77	0.68	0.48
hGH	22	2.38	5.27	0.033^*e*^	(48)	0.00838	2.77	0.71	0.52
hGH	22	2.38	5.27	200^*f*^	(60)	50.8^*f*^	2.77	0.48	0.69
hGH	22	2.38	5.27	1.30^*g*^	(49)	0.00708	2.77	0.40	0.56
EPO	30.4	2.70	8.75	0.000313^*h*^	(51)	0.000280	10.98	1.24	0.84
EPO	30.4	2.70	8.75	0.000938^*h*^	(52)	0.000839	10.97	1.08	0.61
EPO	30.4	2.70	8.75	0.00188^*h*^	(52)	0.00201	10.97	1.25	0.61
EPO	30.4	2.70	8.75	0.00188^*h*^	(50)	0.00201	10.97	0.91	0.84
EPO	30.4	2.70	8.75	0.00281^*h*^	(50)	0.00391	10.96	0.63	0.84
EPO	30.4	2.70	8.75	0.00375^*h*^	(50)	0.00494	10.97	0.70	0.33
EPO	30.4	2.70	8.75	0.00563^*h*^	(50)	0.00867	10.97	0.78	0.65
EPO	30.4	2.70	8.75	0.0075^*h*^	(50)	0.0117	10.96	0.84	0.37
EPO	30.4	2.70	8.75	0.00844^*h*^	(50)	0.0153	10.96	0.77	0.65
EPO	30.4	2.70	8.75	0.0113^*h*^	(50)	0.0233	10.96	0.75	0.30
EPO	30.4	2.70	8.75	0.015^*h*^	(50)	0.0373	10.98	0.95	0.65
EPO	30.4	2.70	8.75	0.000188^*h*^	(53)	0.000168	10.98	1.28	0.92
EPO	30.4	2.70	8.75	0.000375^*h*^	(53)	0.000335	10.98	1.15	0.92
EPO	30.4	2.70	8.75	0.000625^*h*^	(53)	0.00559	10.96	1.24	0.91
Albumin	67	3.55	5.67	100^*i*^	(27)	9.35^*i*^	42.10	1.18	0.88
Tralokinumab	144	5.00	UK	150^*c*^	(54)	10.9	62.53	0.64	0.52
Tralokinumab	144	5.00	UK	300^*c*^	(54)	21.7	62.53	0.60	0.52
Etanercept	150	5.08	7.89	25^*c*^	(55)	1.45	41.48	0.99	0.81
Etanercept	150	5.08	7.89	50^*c*^	(56)	2.90	41.62	0.76	0.80
Etanercept	150	5.08	7.89	25^*c*^	(57)	1.45	41.62	1.24	0.44
Etanercept	150	5.08	7.89	10^*c*^	(58)	0.580	41.62	1.44	1.16
Adalimumab	144	5.002	8.25	40^*c*^	Prescribing Info	3.12	63.68	0.66	0.49
Omalizumab	145	5.01	7.03	150^*c*^	(59)	11.8	70.78	0.78	0.59
Omalizumab	145	5.01	7.03	300^*c*^	(59)	23.5	70.78	0.80	0.59
*N*								54	54
Mean								1.06	0.78
*N* within 0.80–1.25								25	17
*N* within 2-fold								50	46

*hGH* human growth hormone, *IGF*-*1* insulin like growth factor 1, *IL* interleukin, *UK* unknown

^*a*^Dose in units of mg/m^2^ converted from units of IU/m^2^ (0.343 IU/pg (40))

^*b*^Dose in units of mg converted from units of IU (4 × 10^6^ IU/mg (61))

^*c*^Dose in units of mg

^*d*^Dose in units of mIU and *C*_max_ in units of mIU/L

^*e*^Dose converted from units of IU/kg (0.33 IU/mg (49))

^*f*^Dose in units of mIU/kg and *C*_max_ in units of mIU/L

^*g*^Dose in units of mg/m^2^ converted from units of IU/m^2^ (0.33 IU/mg (49))

^*h*^Dose converted from units of IU/kg (160,000 IU/mg)

^*i*^Dose and *C*_max_ in units of % dose

## DISCUSSION

In the current study, a whole-body PBPK model has been developed to describe the tissue distribution and SC absorption rate of TPs. Movement of TPs within the model is based on the 2-pore hypothesis (10), with hydrodynamic radius being the only drug-specific parameter used to predict the rate of absorption and the extent of tissue distribution. Use of the 2-pore model will have minimal impact for the prediction of mAb distribution compared to previously published models where distribution is described by convection alone. For smaller TPs, where diffusion through endothelial pores may have a larger contribution to distribution, this model potentially offers an advantage over PBPK models considering only convective movement. In addition to the usual physiological data required for PBPK models (organ weights, blood flows etc.), lymph flow and pore sizes in each tissue were needed to describe the disposition of TPs. Obtaining accurate estimates of lymph flow from different organs in humans is challenging as the clinical measurement of lymph flow is an invasive procedure and as such is not usually conducted in healthy individuals. Obtaining reliable estimates of lymphatic flow is also difficult because lymph cannulation may lead to changes in flow, making it difficult to get an estimate of the unperturbed lymph flow (62). In the model developed here, we used physiological estimates of lymph flow for the different tissues. Unsurprisingly, when used in the context of the PBPK model, these lymph flow values in addition to the optimised pore sizes were suitable to accurately capture the steady-state tissue lymph/plasma concentration ratios of TPs with a large size range (Fig. 2). Sensitivity analysis showed that the steady-state lymph/plasma concentration ratios were not sensitive to individual tissue lymph flows, whereas interstitial fluid *t*_max_ was (Supplemental Material Figures 2–3). Although most of the observed lymph/plasma concentration ratio data are taken from animals for those proteins where human data are also available, large interspecies differences are not evident, indicating that the animal data may be suitable to use for model development and validation where human data are lacking.

The SC site part of the model was also developed using experimental data to determine suitable physiological values for the lymph flow, interstitial volume and endothelial pore radii. The resulting model could reasonably predict the systemic *t*_max_ for a wide range of TPs, with one third of the predicted values falling within 0.8–1.25-fold of observed values. Half the simulated *C*_max_ values were within 0.8–1.25-fold of observed values. The reasonable prediction of *C*_max_ is unsurprising as it is not only dependent on the absorption rate but also on bioavailability, which was used as an input parameter to the model. A previous dermal clearance model, also based on the 2-pore hypothesis, used similar values for the radii of small and large pores; 5 and 25 nm, respectively (11), compared to the values used here (5 and 20 nm). The lymph rate values used in the two models were also similar (8 and 18 × 10^−6^/s). Previously published models describing SC absorption of proteins incorporating both lymph and blood absorption rates have generally not accounted for the redistribution of TP from the systemic circulation (35,36) but instead have modelled the SC compartment as an absorption site only. However, extra-vascular distribution of TPs is known to be important; for instance; absorbed trastuzumab molecules have been estimated to circulate through the lymphatic system four to five times on average prior to elimination (63). A recent model accounting for redistribution of TP into the SC site interstitial fluid did not incorporate direct blood absorption (16). An advantage of the current model is that it accounts for potential subsequent redistribution of TP into the interstitial fluid at the SC site following absorption and circulation in the blood, which is a closer representation of the processes that occur *in vivo*. In addition, the model developed here considers direct blood absorption at the SC site, which may be important for smaller TPs (13), and hence should give a more realistic description of SC absorption rate.

The diffusion rate through the interstitial space is dictated by molecular size and physical and electrostatic interaction with the various components of the interstitium (e.g. collagen and glycosaminoglycans) (7,12,64). Decreased distribution at the SC injection site and increased electrostatic interactions are expected for TPs with a positive charge at neutral pH (5,35,65). Several studies have shown delayed SC absorption of positively charged compounds compared to negatively charged molecules of the same molecular size (65,66) and reduced SC bioavailability of mAbs with higher pI values (67). Prediction accuracy of TP *C*_max_ and *t*_max_ was compared with pI for the current dataset; however, no correlation was apparent between the accuracy of predictions and the pI of the TPs (Fig. 5). Unfortunately, the majority of TPs used covered a limited range of pI (5.2 to 8.8), with the exception of IL-11 (pI = 11.2). Therefore, it cannot be confirmed from this analysis if charge has an important influence on TP distribution and absorption rate from the SC site; however, it does not appear to be the main/only cause of the poor prediction of *t*_max_ for certain TPs. Similarly, Mach *et al.* (68) suggested that electrostatic interactions are unlikely to have a major influence on mAb absorption rate and bioavailability unless they have a significantly positive charge and are administered at low concentrations. In addition, *ex vivo* studies have shown that interactions with other charged formulation excipients can prevent marked electrostatic interaction between the TP and the SC tissue (68). For in-depth discussion of the impact of TP chemistry and formulation impacts on SC absorption, please see Kinunnen *et al.* (65).

The site and depth of SC injection can also influence the local distribution and hence absorption of TPs (12,69–72). The extent of absorption is generally similar between dosing sites, but the rate of absorption may vary (5). For example, following SC administration of 1 mg/kg rituximab to the foot or back of rats, *t*_max_ was 12 h and 4.6 days, respectively, whereas bioavailability was ~70% for both administration sites (72). In contrast, no marked differences in the exposure and bioavailability of golimumab were apparent following SC administration in the upper thigh, arm or abdomen of healthy adults (73). Regional differences in blood and/or lymph flow are thought to contribute to this site-specific variation in absorption rate (12,71). In addition, exercise, heating and rubbing increase local lymph flow rate (5,74,75) and hence may affect distribution rate, *t*_max_ and bioavailability. Other factors can also influence SC absorption and bioavailability of TPs, including those related to the formulation (pH, viscosity, osmolality, aggregation, excipients), administration (dose level, volume) and patient (disease) (5,12,50,65,68,72,73). Unfortunately, information relating to formulations, dose site and volumes and patient temperature/movement were generally unavailable for the studies used to supply the observed data herein. Therefore the impact of these factors on prediction of SC absorption rate using the PBPK model could not be investigated.

In this study, a PBPK model was developed to incorporate physiological data and aid mechanistic understanding and prediction of the rate of SC absorption. However, a general trend for underprediction of *t*_max_ was observed when using the PBPK model. Lag times of up to 3 h have been included in other models of TP SC absorption to describe the time delay between dose and recovery in central lymph (14,36,45,76–78). Similarly, the radiolabelled IgG data used for model development showed a lag of ~0.5 h between dosing and loss of IgG from the dose site (28–30). In contrast, the required lag time for pegylated EPO was found to be negligible for all species apart from rat in a population PK model (79). Unlike previous models (14,36,45,69,72,80–82), a lag time/delay compartment is not included in the PBPK model. It is currently unknown whether the lymphatic transport or movement through the interstitial fluid at the SC site is the rate-limiting step in SC absorption (5). Multiple factors can contribute to the observed delay, as described above, and a mechanistic description incorporating all these processes is not available. However, as the observed, *t*_max_ for most TPs is >4 h, and for proteins larger than albumin, *t*_max_ is >50 h, incorporating an empirical lag time of ~1 h to account for the transfer of TP from injection site to interstitial space/lymphatics would have minimal impact on the prediction accuracy of *t*_max_.

Transcytosis of mAbs across endothelial cells *via* binding to FcRn may allow direct access to blood at the SC site. However, evidence for the importance of this process to the rate of SC absorption is contradictory. SC dosing of mAbs in FcRn knockout mice showed SC bioavailability was 3-fold lower than in that wild-type mice (31). Correspondingly, increasing FcRn affinity at pH 6 improved bioavailability in mice (83). In contrast, data from cynomolgus monkeys showed no improvement in mAb bioavailability and a decrease in absorption rate when FcRn binding at pH 6 was increased while maintaining no direct binding to FcRn at pH 7.4 (84). Similarly, modelling efforts to describe SC absorption of mAbs incorporating the FcRn contribution have given conflicting results. Predicted absorption *via* FcRn-mediated trancytosis when using such models suggests this route provides <91% (85), 32% (63) or ~0% (14) of the overall systemic absorption in rats, mice and humans, respectively. In addition, lymph flow rate was shown to be the only influential factor for predicted *t*_max_ (14). Although FcRn may have a role in the protection of mAbs from catabolism at the SC site, and hence bioavailability, it is uncertain whether FcRn-mediated transcytosis is an important route of mAb absorption (31,83). Using the PBPK model herein, no clear correlation was observed between TP type and the accuracy of *t*_max_ predictions. This indicates that the model is suitable for both smaller TPs and mAbs and suggests it is not necessary to include the FcRn recycling mechanism in the current model, although this would need to be considered if efforts were made to mechanistically predict bioavailability of mAbs.

One limitation of the current model is the inability to mechanistically predict bioavailability and hence the requirement for a measured value. Bioavailability is highly variable between subjects and species, with no correlation to molecular size (5,86). For mAbs, which all have a similar molecular size, bioavailability usually ranges from 50 to 100% (31). Incomplete absorption is due to degradation and/or catabolism at the SC site and potentially within the lymphatic system prior to the TP entering the systemic circulation. No difference in the fraction of EPO dose recovered in peripheral and central lymph was reported in cannulated sheep, suggesting no loss of EPO during lymph transit from the dosing site to the central blood stream (36) However, the opposite was found for human growth hormone (87). In addition to pre-systemic nonspecific clearance, target-mediated drug disposition (TMDD) may contribute to incomplete bioavailability (88) of TPs when the molecular target of the protein are located within the lymphatic system or at the SC site, leading to dose-dependent bioavailability. For example, dose-dependent bioavailability has been observed for hGH and EPO (47,50). In addition, consideration of TMDD in the model may improve the predictions for TPs where systemic clearance changes with dose due to target receptor saturation. One advantage of the model structure used here is the ability to predict interstitial concentration, which represents the driving concentration of TMDD for membrane-bound target receptors located on the cell surface. Use of the total tissue concentration to model TMDD in such cases would underestimate the concentration at the receptor site as the TPs often do not distribute into the tissue cells themselves and hence total tissue concentration will be lower than Ci.

The current PBPK model could be expanded to incorporate the FcRn recycling mechanism for mAbs and also TMDD models. However, further work is required to understand pre-systemic catabolism/degradation before these processes can be predicted with a true bottom-up approach. *In vitro* incubation of TPs with SC tissue homogenate, lymph and blood (89,90) may help to inform such models in the future. With increased understanding of these processes and their importance for SC bioavailability, the current PBPK model can be expanded to mechanistically describe bioavailability and absorption rate. In addition, the impact of factors such as TP charge, formulation and patient characteristics on SC absorption rate may be incorporated. The current model is however a suitable starting point for bottom-up prediction of the rate of SC absorption. Another important consideration when predicting drug kinetics is obtaining an accurate representation of the variability within a given population (91). Although the current study focuses on predictions for an average person, future work is intended to explore the prediction of population variability in absorption following SC dosing.

## CONCLUSION

A mechanistic whole-body PBPK model has been developed to predict absorption rate of TPs following SC dosing *via* both direct diffusion through capillaries into blood and through lymphatic absorption. The model provided reasonable prediction of SC absorption using a bottom-up approach based on TP molecular size as the model input. One third to half the *C*_max_ and *t*_max_ predictions fell within 0.8–1.25-fold of the observed values. Although a general trend for underprediction of *t*_max_ was observed, no correlation with molecular size or pI was apparent. Further enhancement in the future to include mechanistic prediction of distribution at the injection site and through the interstitial space as well as pre-systemic elimination will allow a true bottom-up approach for prediction of TP SC absorption.

## Electronic Supplementary Material

Below is the link to the electronic supplementary material.

ESM 1 (DOCX 673 kb)

## References

[1] Walsh G (2010). Biopharmaceutical benchmarks 2010. Nat Biotechnol.

[2] Dostalek M, Gardner I, Gurbaxani BM, Rose RH, Chetty M (2013). Pharmacokinetics, pharmacodynamics and physiologically-based pharmacokinetic modelling of monoclonal antibodies. Clin Pharmacokinet.

[3] Keizer R, Huitema AR, Schellens JM, Beijnen J (2010). Clinical pharmacokinetics of therapeutic monoclonal antibodies. Clin Pharmacokinet.

[4] Lobo ED, Hansen RJ, Balthasar JP (2004). Antibody pharmacokinetics and pharmacodynamics. J Pharm Sci.

[5] Richter WF, Bhansali SG, Morris ME (2012). Mechanistic determinants of biotherapeutics absorption following SC administration. AAPS J.

[6] Aukland K, Reed RK (1993). Interstitial-lymphatic mechanisms in the control of extracellular fluid volume. Physiol Rev.

[7] Swartz MA (2001). The physiology of the lymphatic system. Adv Drug Deliv Rev.

[8] Frost GI (2007). Recombinant human hyaluronidase (rHuPH20): an enabling platform for subcutaneous drug and fluid administration. Expert Opin Drug Deliv.

[9] Swartz MA, Fleury ME (2007). Interstitial flow and its effects in soft tissues. Annu Rev Biomed Eng.

[10] Rippe B, Haraldsson B (1987). Fluid and protein fluxes across small and large pores in the microvasculature. Application of two-pore equations. Acta Physiol Scand.

[11] Ibrahim R, Nitsche JM, Kasting GB (2012). Dermal clearance model for epidermal bioavailability calculations. J Pharm Sci.

[12] Porter CJH, Charman SA (2000). Lymphatic transport of proteins after subcutaneous administration. J Pharm Sci.

[13] Supersaxo A, Hein WR, Steffen H (1990). Effect of molecular weight on the lymphatic absorption of water-soluble compounds following subcutaneous administration. Pharm Res.

[14] Zhao L, Ji P, Li Z, Roy P, Sahajwalla CG (2013). The antibody drug absorption following subcutaneous or intramuscular administration and its mathematical description by coupling physiologically based absorption process with the conventional compartment pharmacokinetic model. J Clin Pharmacol.

[15] Kagan L (2014). Pharmacokinetic modeling of the subcutaneous absorption of therapeutic proteins. Drug Metab Dispos.

[16] Offman E, Edginton A. A PBPK workflow for first-in-human dose selection of a subcutaneously administered pegylated peptide. J Pharmacokinet Pharmacodyn. 2015;1–16. doi:10.1007/s10928-015-9406-4. 10.1007/s10928-015-9406-4 25650156

[17] Baxter LT, Zhu H, Mackensen DG, Butler WF, Jain RK (1995). Biodistribution of monoclonal antibodies: scale-up from mouse to human using a physiologically based pharmacokinetic model. Cancer Res.

[18] Baxter LT, Zhu H, Mackensen DG, Jain RK (1994). Physiologically based pharmacokinetic model for specific and nonspecific monoclonal antibodies and fragments in normal tissues and human tumor xenografts in nude mice. Cancer Res.

[19] ICRP (2002). Basic anatomical and physiological data for use in radiological protection: reference values. ICRP publication 89. Ann ICRP.

[20] Jamei M, Marciniak S, Feng K, Barnett A, Tucker G, Rostami-Hodjegan A (2009). The Simcyp® population-based ADME simulator. Expert Opin Drug Metab Toxicol.

[21] Jamei M, Bajot F, Neuhoff S, Barter Z, Yang J, Rostami-Hodjegan A (2014). A mechanistic framework for in vitro–in vivo extrapolation of liver membrane transporters: prediction of drug–drug interaction between rosuvastatin and cyclosporine. Clin Pharmacokinet.

[22] Guyton AC (1973). Circulatory physiology: cardiac output and its regulation.

[23] Levy MN, Pappano AJ (2007). Cardiovascular physiology.

[24] Pappano A, Koeppen B, Stanton B (2008). Properties of the vasculature. Berne and levy physiology.

[25] Koeppen B, Stanton B (2008). Berne and levy physiology.

[26] Taylor A, Granger D, Renkin E, Michel C (1984). Exchange of macromolecules across the microcirculation. Handbook of physiology: the cardiovascular system microcirculation.

[27] Hollander W, Reilly P, Burrows BA (1961). Lymphatic flow in human subjects as indicated by the disappearance of 1-131-labeled albumin from the subcutaneous tissue. J Clin Invest.

[28] Stanton AWB, Modi S, Mellor RH, Peters AM, Svensson WE, Levick JR (2006). A quantitative lymphoscintigraphic evaluation of lymphatic function in the swollen hands of women with lymphoedema following breast cancer treatment. Clin Sci.

[29] Stanton AW, Svensson WE, Mellor RH, Peters AM, Levick JR, Mortimer PS (2001). Differences in lymph drainage between swollen and non-swollen regions in arms with breast-cancer-related lymphoedema. Clin Sci.

[30] Pain SJ, Barber RW, Solanki CK, Ballinger JR, Britton TB, Mortimer PS (2005). Short-term effects of axillary lymph node clearance surgery on lymphatic physiology of the arm in breast cancer. J Appl Physiol.

[31] Wang W, Wang EQ, Balthasar JP (2008). Monoclonal antibody pharmacokinetics and pharmacodynamics. Clin Pharmacol Ther.

[32] Levitt DG (2003). The pharmacokinetics of the interstitial space in humans. BMC Clin Pharmacol.

[33] Poulsen HL (1973). Subcutaneous interstitial fluid albumin concentration in long-term diabetes mellitus. Scand J Clin Lab Invest.

[34] Poulsen HL (1974). Interstitial fluid concentrations of albumin and immunoglobulin G in normal men. Scand J Clin Lab Invest.

[35] McLennan D, Porter CH, Edwards G, Heatherington A, Martin S, Charman S (2006). The absorption of darbepoetin alfa occurs predominantly via the lymphatics following subcutaneous administration to sheep. Pharm Res.

[36] McLennan DN, Porter CJH, Edwards GA, Martin SW, Heatherington AC, Charman SA (2005). Lymphatic absorption is the primary contributor to the systemic availability of epoetin alfa following subcutaneous administration to sheep. J Pharmacol Exp Ther.

[37] Grahnen A, Kastrup K, Heinrich U, Gourmelen M, Preece MA, Vaccarello MA (1993). Pharmacokinetics of recombinant human insulin-like growth factor I given subcutaneously to healthy volunteers and to patients with growth hormone receptor deficiency. Acta Paediatr Suppl.

[38] Fouque D, Peng SC, Kopple JD (1995). Pharmacokinetics of recombinant human insulin-like growth factor-1 in dialysis patients. Kidney Int.

[39] Wilton P, Sietnieks A, Gunnarsson R, Berger L, Grahnen A (1991). Pharmacokinetic profile of recombinant human insulin-like growth factor I given subcutaneously in normal subjects. Acta Paediatr Scand Suppl.

[40] Kirchner GI, Franzke A, Buer J, Beil W, Probst-Kepper M, Wittke F (1998). Pharmacokinetics of recombinant human interleukin-2 in advanced renal cell carcinoma patients following subcutaneous application. Br J Clin Pharmacol.

[41] Piscitelli SC, Wells MJ, Metcalf JA, Baseler M, Stevens R, Davey RT (1996). Pharmacokinetics and pharmacodynamics of subcutaneous interleukin-2 in HIV-infected patients. Pharmacotherapy.

[42] Yang B-B, Baughman S, Sullivan JT (2003). Pharmacokinetics of anakinra in subjects with different levels of renal function. Clin Pharmacol Ther.

[43] Chakraborty A, Blum RA, Mis SM, Cutler DL, Jusko WJ (1999). Pharmacokinetic and adrenal interactions of IL-10 and prednisone in healthy volunteers. J Clin Pharmacol.

[44] Radwanski E, Chakraborty A, Van Wart S, Huhn RD, Cutler DL, Affrime MB (1998). Pharmacokinetics and leukocyte responses of recombinant human interleukin-10. Pharm Res.

[45] Huhn RD, Radwanski E, Gallo J, Affrime MB, Sabo R, Gonyo G (1997). Pharmacodynamics of subcutaneous recombinant human interleukin-10 in healthy volunteers. Clin Pharmacol Ther.

[46] Aoyama K, Uchida T, Takanuki F, Usui T, Watanabe T, Higuchi S (1997). Pharmacokinetics of recombinant human interleukin-11 (rhIL-11) in healthy male subjects. Br J Clin Pharmacol.

[47] Janssen YJH, Frölich M, Roelfsema F (1999). The absorption profile and availability of a physiological subcutaneously administered dose of recombinant human growth hormone (GH) in adults with GH deficiency. Br J Clin Pharmacol.

[48] Laursen T, Grandjean B, Jorgensen JO, Christiansen JS (1996). Bioavailability and bioactivity of three different doses of nasal growth hormone (GH) administered to GH-deficient patients: comparison with intravenous and subcutaneous administration. Eur J Endocrinol.

[49] Zeisel HJ, von Petrykowski W, Wais U (1992). Pharmacokinetics and short-term metabolic effects of mammalian cell-derived biosynthetic human growth hormone in man. Horm Res.

[50] Ramakrishnan R, Cheung WK, Wacholtz MC, Minton N, Jusko WJ (2004). Pharmacokinetic and pharmacodynamic modeling of recombinant human erythropoietin after single and multiple doses in healthy volunteers. J Clin Pharmacol.

[51] Salmonson T, Danielson BG, Wikström B (1990). The pharmacokinetics of recombinant human erythropoietin after intravenous and subcutaneous administration to healthy subjects. Br J Clin Pharmacol.

[52] McMahon F, Vargas R, Ryan M, Jain A, Abels R, Perry B (1990). Pharmacokinetics and effects of recombinant human erythropoietin after intravenous and subcutaneous injections in healthy volunteers. Blood.

[53] Sans T, Joven J, Vilella E, Masdeu G, Farrè M (2000). Pharmacokinetics of several subcutaneous doses of erythropoietin: potential implications for blood transfusion. Clin Exp Pharmacol Physiol.

[54] Oh CK, Faggioni R, Jin F, Roskos LK, Wang B, Birrell C (2010). An open-label, single-dose bioavailability study of the pharmacokinetics of CAT-354 after subcutaneous and intravenous administration in healthy males. Br J Clin Pharmacol.

[55] Korth-Bradley JM, Rubin AS, Hanna RK, Simcoe DK, Lebsack ME (2000). The pharmacokinetics of etanercept in healthy volunteers. Ann Pharmacother.

[56] Sullivan JT, Ni L, Sheelo C, Salfi M, Peloso PM (2006). Bioequivalence of liquid and reconstituted lyophilized etanercept subcutaneous injections. J Clin Pharmacol.

[57] Yi S, Kim S, Park M-K, Yoon S, Cho J-Y, Lim K (2012). Comparative pharmacokinetics of HD203, a biosimilar of etanercept, with marketed etanercept (Enbrel ®): a double-blind, single-dose, crossover study in healthy volunteers. BioDrugs.

[58] Zhou H (2005). Clinical pharmacokinetics of etanercept: a fully humanized soluble recombinant tumor necrosis factor receptor fusion protein. J Clin Pharmacol.

[59] Rivière G, Yeh C, Reynolds C, Brookman L, Kaiser G (2011). Bioequivalence of a novel omalizumab solution for injection compared with the standard lyophilized powder formulation. J Bioequiv Availab.

[60] Ho KY, Weissberger AJ, Stuart MC, Day RO, Lazarus L (1989). The pharmacokinetics, safety and endocrine effects of authentic biosynthetic human growth hormone in normal subjects. Clin Endocrinol (Oxf).

[61] Konrad MW, Hemstreet G, Hersh EM, Mansell PWA, Mertelsmann R, Kolitz JE (1990). Pharmacokinetics of recombinant interleukin 2 in humans. Cancer Res.

[62] Porter CJH, Charman WN (2001). Intestinal lymphatic drug transport: an update. Adv Drug Deliv Rev.

[63] Dahlberg AM, Kaminskas LM, Smith A, Nicolazzo JA, Porter CJH, Bulitta JB (2013). The lymphatic system plays a major role in the intravenous and subcutaneous pharmacokinetics of trastuzumab in rats. Mol Pharm.

[64] Shi S (2014). Biologics: an update and challenge of their pharmacokinetics. Curr Drug Metab.

[65] Kinnunen HM, Mrsny RJ (2014). Improving the outcomes of biopharmaceutical delivery via the subcutaneous route by understanding the chemical, physical and physiological properties of the subcutaneous injection site. J Control Release.

[66] Reddy ST, Berk DA, Jain RK, Swartz MA (2006). A sensitive in vivo model for quantifying interstitial convective transport of injected macromolecules and nanoparticles. J Appl Physiol.

[67] Zheng Y, Tesar DB, Benincosa L, Birnböck H, Boswell CA, Bumbaca D (2012). Minipig as a potential translatable model for monoclonal antibody pharmacokinetics after intravenous and subcutaneous administration. MAbs.

[68] Mach H, Gregory SM, Mackiewicz A, Mittal S, Lalloo A, Kirchmeier M (2011). Electrostatic interactions of monoclonal antibodies with subcutaneous tissue. Ther Deliv.

[69] Kagan L, Mager DE (2013). Mechanisms of subcutaneous absorption of rituximab in rats. Drug Metab Dispos.

[70] Beshyah SA, Anyaoku V, Niththyananthan R, Sharp P, Johnston DG (1991). The effect of subcutaneous injection site on absorption of human growth hormone: abdomen versus thigh. Clin Endocrinol (Oxf).

[71] Jensen JD, Jensen LW, Madsen JK (1994). The pharmacokinetics of recombinant human erythropoietin after subcutaneous injection at different sites. Eur J Clin Pharmacol.

[72] Kagan L, Turner M, Balu-Iyer S, Mager D (2012). Subcutaneous absorption of monoclonal antibodies: role of dose, site of injection, and injection volume on rituximab pharmacokinetics in rats. Pharmac Res.

[73] Xu Z, Wang Q, Zhuang Y, Frederick B, Yan H, Bouman-Thio E (2010). Subcutaneous bioavailability of golimumab at 3 different injection sites in healthy subjects. J Clin Pharmacol.

[74] Olszewski W, Engeset A, Icger PM, Sokolowski J, Theodorsen L (1977). Flow and composition of leg lymph in normal Men during venous stasis, muscular activity and local hyperthermia. Acta Physiol Scand.

[75] Havas E, Parviainen T, Vuorela J, Toivanen J, Nikula T, Vihko V (1997). Lymph flow dynamics in exercising human skeletal muscle as detected by scintography. J Physiol.

[76] Gopalakrishnan M, Suarez S, Hickey A, Gobburu J (2005). Population pharmacokinetic–pharmacodynamic modeling of subcutaneous and pulmonary insulin in rats. J Pharmacokinet Pharmacodyn.

[77] Olsson-Gisleskog P, Jacqmin P, Perez-Ruixo J (2007). Population pharmacokinetics meta-analysis of recombinant human erythropoietin in healthy subjects. Clin Pharmacokinet.

[78] Fang Y, Li L-j, Wang R, Huang F, Song H-f, Tang Z-m (2010). Population pharmacokinetics of rhTNFR-Fc in healthy Chinese volunteers and in Chinese patients with ankylosing spondylitis. Acta Pharmacol Sin.

[79] Jolling K, Perez Ruixo JJ, Hemeryck A, Vermeulen A, Greway T (2005). Mixed-effects modelling of the interspecies pharmacokinetic scaling of pegylated human erythropoietin. Eur J Pharm Sci.

[80] Mager D, Jusko W (2002). Receptor-mediated pharmacokinetic/pharmacodynamic model of interferon-β 1a in humans. Pharm Res.

[81] Mager DE, Neuteboom B, Efthymiopoulos C, Munafo A, Jusko WJ (2003). Receptor-mediated pharmacokinetics and pharmacodynamics of interferon-β1a in monkeys. J Pharmacol Exp Ther.

[82] Segrave AM, Mager DE, Charman SA, Edwards GA, Porter CJH (2004). Pharmacokinetics of recombinant human leukemia inhibitory factor in sheep. J Pharmacol Exp Ther.

[83] Deng R, Meng YG, Hoyte K, Lutman J, Lu Y, Iyer S (2012). Subcutaneous bioavailability of therapeutic antibodies as a function of FcRn binding affinity in mice. MAbs.

[84] Datta-Mannan A, Witcher DR, Lu J, Wroblewski VJ (2012). Influence of improved FcRn binding on the subcutaneous bioavailability of monoclonal antibodies in cynomolgus monkeys. MAbs.

[85] Kagan L, Zhao J, Mager D (2014). Interspecies pharmacokinetic modeling of subcutaneous absorption of rituximab in mice and rats. Pharm Res.

[86] McDonald TA, Zepeda ML, Tomlinson MJ, Bee WH, Ivens IA (2010). Subcutaneous administration of biotherapeutics: current experience in animal models. Curr Opin Mol Ther.

[87] Charman SA, Segrave AM, Edwards GA, Porter CJH (2000). Systemic availability and lymphatic transport of human growth hormone administered by subcutaneous injection. J Pharm Sci.

[88] Mager DE, Jusko WJ (2001). General pharmacokinetic model for drugs exhibiting target-mediated drug disposition. J Pharmacokinet Pharmacodyn.

[89] Wang W, Chen N, Shen X, Cunningham P, Fauty S, Michel K (2012). Lymphatic transport and catabolism of therapeutic proteins after subcutaneous administration to rats and dogs. Drug Metab Dispos.

[90] Zou Y, Bateman TJ, Adreani C, Shen X, Cunningham PK, Wang B (2013). Lymphatic absorption, metabolism, and excretion of a therapeutic peptide in dogs and rats. Drug Metab Dispos.

[91] Jamei M, Dickinson GL, Rostami-Hodjegan A (2009). A framework for assessing inter-individual variability in pharmacokinetics using virtual human populations and integrating general knowledge of physical chemistry, biology, anatomy, physiology and genetics: a tale of ‘bottom-up’ vs ‘top-down’ recognition of covariates. Drug Metab Pharmacokinet.

