# Supplementary material for: A Bottom-Up Whole-Body Physiologically Based Pharmacokinetic Model to Mechanistically Predict Tissue Distribution and the Rate of Subcutaneous Absorption of Therapeutic Proteins
Source: AAPS J. 2015 Sep 25;18(1):156–70. doi: 10.1208/s12248-015-9819-4 (PMC6890583; doi:10.1208/s12248-015-9819-4)
Supplement: Supplementary file 1 — (DOCX 673 kb) [file 12248_2015_9819_MOESM1_ESM.docx]

A Bottom-up Whole Body Physiologically Based Pharmacokinetic Model to Mechanistically Predict Tissue Distribution and the Rate of Subcutaneous Absorption of Therapeutic Proteins

Katherine L Gill, Iain Gardner, Linzhong Li and Masoud Jamei

**Supplemental Material**

**Reference for lymph flow values presented in Table I:** **System parameters used in the whole body PBPK model for describing the pharmacokinetics of therapeutic proteins.**

Auckland, Reed. Interstitial-lymphatic mechanisms in the control of extracellular fluid volume. Physiol Rev. 1993; 73, 1-78.

Bauer, Christ, Gamble. Can lymphatic drainage be measured non-invasively in human limbs, using plethysmography? Clin Sci. 2004; 106, 627-633. doi: 10.1042/CS20030314.

Ewerth, Bjorkhem, Einarsson, Ost. Lymphatic transport of bile acids in man. J of Lipid Res. 1982;23, 1183-1186.

Fanous, Phillips, Windsor. Mesenteric lymph: The bridge to future management of critical illness. J Pancreas (Online). 2007; 8, 374-399.

Guyton AC. Circulatory Physiology: Cardiac Output and Its Regulation.1973.

Holliday. Extracellular fluid and its proteins: dehydration, shock, and recovery. Pediatr Nephrol. 1999; 13, 989

Levick JR. An Introduction to Cardiovascular Physiology. Fifth Edition. CRC Press: Boca Raton, FL. 2010.

Malbrain, Pelosi, De laet, Lattuada, Hedenstierna. Lymphatic drainage between thorax and abdomen: please take good care of this well-performing machinery… Acta Clin Belg Suppl. 2007; 62, 152-161.

Mallick, Bodenham. Disorders of the lymph circulation: their relevance to anaesthesia and intensive care. Br J Anesthesia. 2003; 91, 265-72. doi: 10.1093/bja/aeg155.

Miller, Michel, Nanjee, Olszewski, Miller, Hazell et al. Secretion of adipokines by human adipose tissue in vivo: partitioning between capillary and lymphatic transport. Am J Physiol Endocrinol Metab. 2011;301, 659–667. doi: 10.1152/ajpendo.00058.

Miserocchi. Physiology and pathophysiology of pleural fluid turnover. Eur Respir J. 1997; 10, 219-225. doi: 10.1183/09031936.97.10010219.

Olszewski, Pazdur, Kubasiewicz, Zaleska, Cooke, Miller. Lymph draining from foot joints in rheumatoid arthritis provides insight into local cytokine and chemokine production and transport to lymph nodes. Arthritis Rheum. 2001; 44, 541-549. doi: 10.1002/1529-0131.

Porter, Charman. Lymphatic transport of proteins after subcutaneous administration. J Pharm Sci. 2000; 89, 297-310. doi: 10.1002/(SICI)1520-6017.

Reddy. Lymph circulation: Physiology, pharmacology, and biomechanics. Crit Rev Biomed Eng. 1986; 14, 45-91.

Renkin. Some consequences of capillary permeability to macromolecules: Starling's hypothesis reconsidered. Am J Physiolo. 1986; 86, H706-H710.

Renkin, Wiig. Limits to steady-state lymph flow rates derived from plasma-to-tissue uptake measurements. Microvasc Res. 1994; 47, 318-328. doi: 10.1006/mvre.1994.1025.

Rössler, Fink, Goswami, Batzel. Modeling of hyaluronan clearance with application to estimation of lymph flow. Physiol Meas. 2011; 32, 1213–1238. doi: 10.1088/0967-3334/32/8/014.

Samanta, Saini, Chhuttani, Patra, Vashista, Datta. Thoracic duct and hepatic lymph in idiopathic portal hypertension. Gut. 1974; 15, 903-906. doi: 10.1136/gut.15.11.903.

Skandalakis, Skandalakis, Skandalakis. Anatomy of the lymphatics. Surg Oncol Clin N Am. 2007; 16, 1-16. doi: 10.1016/j.soc.2006.10.006.

Swartz. The physiology of the lymphatic system. Adv Drug Deliv Rev. 2001; 50, 3–20. doi: 10.1016/S0169-409X(01)00150-8.

–995.

Yoffey, Courtice. Lymphatics, Lymph and Lymphomyeloid Complex. Academic Press: New York. 1970.

**Reference for pore size and ratio values prior to optimisation presented in Table I:** **System parameters used in the whole body PBPK model for describing the pharmacokinetics of therapeutic proteins.**

Simionescu, Gafencu, Antohe. Transcytosis of plasma macromolecules in endothelial cells: a cell biological survey. Microsc Res Tech. 2002; 57(5), 269-88. doi: 10.1002/jemt.10086.

Taylor, Granger. In Handbook of Physiology. The Cardiovascular System. Microcirculation, Bethesda, MD: Am Physiol Soc, Sect 2, Part 1, Chapt 11: 467-520. 1984.

McNamee, Staub. Pore models of sheep lung microvascular barrier using new data on protein tracers. Microvasc Res. 1979; 18, 229-244.

Renkin. Ambiguities and errors in evaluation of capillary pore sizes. (Letter to the editor) Am. J. Physiol. 1980; 240 (Heart Circ. Physiol. 9), H145-H146.

Michel, Curry. Microvascular permeability. Physiol Rev. 1999; 79(3), 703-61.

Parker, Crain, Grimbert, Rutili, Taylor. Total lung lymph flow and fluid compartmentation in edematous dog lungs. J Appl Physiol Respir Environ Exerc Physiol. 1981; 51(5), 1268-77.

Siflinger-Birnboim, Del Vecchio, Cooper, Blumenstock, Shepard, Malik. Molecular sieving characteristics of the cultured endothelial monolayer. J Cell Physiol. 1987; 132(1), 111-7.

Taylor, Gaar. Estimation of equivalent pore radii of pulmonary capillary and alveolar membranes. Am J Physiol. 1970; 218(4), 1133-40.

**References for Figure 3: Predicted and observed Ci/Cp ratios for proteins with a range of hydrodynamic radii.**

Abuqayyas, Balthasar. Investigation of the role of FcγR and FcRn in mAb distribution to the brain. Mol Pharm. 2013; 10(5) 1505-13 . doi: 10.1021/mp300214k.

Ellmerer, Schaupp, Brunner, Sendlhofer, Wutte, Wach, Pieber. Measurement of interstitial albumin in human skeletal muscle and adipose tissue by open-flow microperfusion. Am J Physiol Endocrinal Metab, 2000 ; 278, E352-6.Garlick, Renkin. Transport of large molecules from plasma to interstitial fluid and lymph in dogs. Am J Physiol. 1970; 219(6):1595-605.

Levitt. The pharmacokinetics of the interstitial space in humans. BMC Clin Pharmacol. 2003; 3 (3: 1-29. doi: 10.1186/1472-6904-3-3.

Miller, Michel, Nanjee, Olszewski, Miller, Hazell et al. Secretion of adipokines by human adipose tissue in vivo: partitioning between capillary and lymphatic transport. Am J Physiol Endocrinol Metab. 2011;301, 659–667. doi: 10.1152/ajpendo.00058.

Olszewski, Pazdur, Kubasiewicz, Zaleska, Cooke, Miller. Lymph draining from foot joints in rheumatoid arthritis provides insight into local cytokine and chemokine production and transport to lymph nodes. Arthritis Rheum. 2001; 44, 541-549. doi: 10.1002/1529-0131.

Parker, Gilchrist, Cartledge. Plasma-lymph exchange and interstitial distribution volumes of charged macromolecules in the lung. J Appl Physiol. 1985 ; 59 (4): 1128-36.

Taylor, Granger. In Handbook of Physiology. The Cardiovascular System. Microcirculation, Bethesda, MD: Am Physiol Soc, Sect 2, Part 1, Chapt 11: 467-520. 1984.

Wang, Wang, Balthasar. Monoclonal antibody pharmacokinetics and pharmacodynamics. Clin Pharmacol Ther. 2008; 84(5) 548-58. doi: 10.1038/clpt.2008.

**Supplemental Table I: Input data used for prediction of C_max_ and t_max_ following subcutaneous dosing of therapeutic proteins with a range of sizes.**

| **Protein** | **Hydrodynamic Radius (nm)** | **Clearance (L/h)** | **Reference** | **Bioavailability** | **Reference** | **Dose (mg/kg)** | **Reference** |
| --- | --- | --- | --- | --- | --- | --- | --- |
| IGF-1 | 1.56 | 1.02 | Grahnen et al., (1993) | 1.00 | Grahnen et al., (1993) | 0.040 | Grahnen et al., (1993) |
| IGF-1 | 1.56 | 1.02 | Grahnen et al., (1993) | 1.00 | Grahnen et al., (1993) | 0.080 | Grahnen et al., (1993) |
| IGF-1 | 1.56 | 1.02 | Grahnen et al., (1993) | 1.00 | Grahnen et al., (1993) | 0.050 | Fouque et al., (1995) |
| IGF-1 | 1.56 | 1.02 | Grahnen et al., (1993) | 1.00 | Grahnen et al., (1993) | 0.100 | Fouque et al., (1995) |
| IGF-1 | 1.56 | 1.02 | Grahnen et al., (1993) | 1.00 | Grahnen et al., (1993) | 0.040 | Wiltern et al., (1991) |
| IGF-1 | 1.56 | 1.02 | Grahnen et al., (1993) | 1.00 | Grahnen et al., (1993) | 0.080 | Wiltern et al., (1991) |
| IL-2 | 2.07 | 8.48 ^j^ | See footnotes | 0.65 | Piscitelli et al., (1996) | 0.0292 ^a^ | Kirchner et al., (1998) |
| IL-2 | 2.07 | 8.48 ^j^ | See footnotes | 0.65 | Piscitelli et al., (1996) | 0.0583 ^a^ | Kirchner et al., (1998) |
| IL-2 | 2.07 | 8.48 ^j^ | See footnotes | 0.65 | Piscitelli et al., (1996) | 3.00 ^b^ | Piscitelli et al., (1996) |
| IL-2 | 2.07 | 8.48 ^j^ | See footnotes | 0.65 | Piscitelli et al., (1996) | 3.75 ^b^ | Piscitelli et al., (1996) |
| IL-2 | 2.07 | 8.48 ^j^ | See footnotes | 0.65 | Piscitelli et al., (1996) | 4.50 ^b^ | Piscitelli et al., (1996) |
| Anakinra | 2.16 | 8.22 | Yang et al., (2003) | 0.872 | Yang et al., (2003) | 100 ^c^ | Yang et al., (2003) |
| IL-10 | 2.23 | 4.17 ^k^ | See footnotes | 0.42 | Radwanski et al., (1998) | 0.008 | Chakraborty et al., (1999) |
| IL-10 | 2.23 | 4.17 ^k^ | See footnotes | 0.42 | Radwanski et al., (1998) | 1.75 ^c^ | Radwanski et al., (1998) |
| IL-10 | 2.23 | 4.17 ^k^ | See footnotes | 0.42 | Radwanski et al., (1998) | 0.001 | Huhn et al., (1997) |
| IL-10 | 2.23 | 4.17 ^k^ | See footnotes | 0.42 | Radwanski et al., (1998) | 0.0025 | Huhn et al., (1997) |
| IL-10 | 2.23 | 4.17 ^k^ | See footnotes | 0.42 | Radwanski et al., (1998) | 0.005 | Huhn et al., (1997) |
| IL-10 | 2.23 | 4.17 ^k^ | See footnotes | 0.42 | Radwanski et al., (1998) | 0.010 | Huhn et al., (1997) |
| IL-10 | 2.23 | 4.17 ^k^ | See footnotes | 0.42 | Radwanski et al., (1998) | 0.025 | Huhn et al., (1997) |
| IL-10 | 2.23 | 4.17 ^k^ | See footnotes | 0.42 | Radwanski et al., (1998) | 0.050 | Huhn et al., (1997) |
| IL-11 | 2.25 | 9.99 | Aoyama et al., (1997) | 0.65 | Aoyama et al., (1997) | 0.003 | Aoyama et al., (1997) |
| IL-11 | 2.25 | 9.99 | Aoyama et al., (1997) | 0.65 | Aoyama et al., (1997) | 0.010 | Aoyama et al., (1997) |
| IL-11 | 2.25 | 9.99 | Aoyama et al., (1997) | 0.65 | Aoyama et al., (1997) | 0.025 | Aoyama et al., (1997) |
| IL-11 | 2.25 | 9.99 | Aoyama et al., (1997) | 0.65 | Aoyama et al., (1997) | 0.050 | Aoyama et al., (1997) |
| hGH | 2.38 | 15.0 ^l^ | Laursen et al., (1996) | 0.67 | Janssen et al., (1999) | 600 ^d^ | Janssen et al., (1999) |
| hGH | 2.38 | 15.0 ^l^ | Laursen et al., (1996) | 0.57 | Janssen et al., (1999) | 1200 ^d^ | Janssen et al., (1999) |
| hGH | 2.38 | 15.0 ^l^ | Laursen et al., (1996) | 0.75 | Janssen et al., (1999) | 1800 ^d^ | Janssen et al., (1999) |
| hGH | 2.38 | 15.0 ^l^ | Laursen et al., (1996) | 0.75 | Janssen et al., (1999) | 0.033 ^e^ | Laursen et al., (1996) |
| hGH | 2.38 | 15.0 ^l^ | Laursen et al., (1996) | 0.75 | Janssen et al., (1999) | 200 ^f^ | Ho et al., (1989) |
| hGH | 2.38 | 15.0 ^l^ | Laursen et al., (1996) | 0.75 | Janssen et al., (1999) | 1.3 ^g^ | Zeisel et al., (1992) |
| EPO | 2.7 | 0.940 | Salmonson et al., (1990) | 0.36 | Salmonson et al., (1990) | 0.000313 ^h^ | Salmonson et al., (1990) |
| EPO | 2.7 | 0.940 | Salmonson et al., (1990) | 0.36 | Salmonson et al., (1990) | 0.000938 ^h^ | McMahon et al., (1990) |
| EPO | 2.7 | 0.940 | Salmonson et al., (1990) | 0.43 | Ramakrishnan et al., (2004) | 0.00188 ^h^ | McMahon et al., (1990) |
| EPO | 2.7 | 0.940 | Salmonson et al., (1990) | 0.43 | Ramakrishnan et al., (2004) | 0.00188 ^h^ | Ramakrishnan et al., (2004) |
| EPO | 2.7 | 0.940 | Salmonson et al., (1990) | 0.56 | Ramakrishnan et al., (2004) | 0.00281 ^h^ | Ramakrishnan et al., (2004) |
| EPO | 2.7 | 0.940 | Salmonson et al., (1990) | 0.53 | Ramakrishnan et al., (2004) | 0.00375 ^h^ | Ramakrishnan et al., (2004) |
| EPO | 2.7 | 0.940 | Salmonson et al., (1990) | 0.62 | Ramakrishnan et al., (2004) | 0.00563 ^h^ | Ramakrishnan et al., (2004) |
| EPO | 2.7 | 0.940 | Salmonson et al., (1990) | 0.63 | Ramakrishnan et al., (2004) | 0.0075 ^h^ | Ramakrishnan et al., (2004) |
| EPO | 2.7 | 0.940 | Salmonson et al., (1990) | 0.73 | Ramakrishnan et al., (2004) | 0.00844 ^h^ | Ramakrishnan et al., (2004) |
| EPO | 2.7 | 0.940 | Salmonson et al., (1990) | 0.83 | Ramakrishnan et al., (2004) | 0.0113 ^h^ | Ramakrishnan et al., (2004) |
| EPO | 2.7 | 0.940 | Salmonson et al., (1990) | 1.00 | Ramakrishnan et al., (2004) | 0.015 ^h^ | Ramakrishnan et al., (2004) |
| EPO | 2.7 | 0.940 | Salmonson et al., (1990) | 0.36 | Salmonson et al., (1990) | 0.000188 ^h^ | Sans et al., (2000) |
| EPO | 2.7 | 0.940 | Salmonson et al., (1990) | 0.36 | Salmonson et al., (1990) | 0.000375 ^h^ | Sans et al., (2000) |
| EPO | 2.7 | 0.940 | Salmonson et al., (1990) | 0.36 | Salmonson et al., (1990) | 0.000625 ^h^ | Sans et al., (2000) |
| Albumin | 3.55 | 0.0462 ^l^ | Hollander et al., (1961) | 1.00 | Assumed | 100 ^i^ | Hollander et al., (1961) |
| Tralokinumab | 4.998 | 0.0131 ^l^ | Oh et al., (2010) | 0.60 | Oh et al., (2010) | 150 ^c^ | Oh et al., (2010) |
| Tralokinumab | 4.998 | 0.0131 ^l^ | Oh et al., (2010) | 0.60 | Oh et al., (2010) | 300 ^c^ | Oh et al., (2010) |
| Etanercept | 5.08 | 0.060 ^l^ | Zhou et al., (2005) | 0.60 | Zhou et al., (2005) | 25 ^c^ | Korth-Bradley et al., (2000) |
| Etanercept | 5.08 | 0.060 ^l^ | Zhou et al., (2005) | 0.60 | Zhou et al., (2005) | 50 ^c^ | Sullivan et al., (2006) |
| Etanercept | 5.08 | 0.060 ^l^ | Zhou et al., (2005) | 0.60 | Zhou et al., (2005) | 25 ^c^ | Yi et al., (2012) |
| Etanercept | 5.08 | 0.060 ^l^ | Zhou et al., (2005) | 0.60 | Zhou et al., (2005) | 10 ^c^ | Zhou et al., (2005) |
| Adalimumab | 5.002 | 0.012 | Prescribing Info | 0.64 | Prescribing Info | 40 ^c^ | Prescribing Info |
| Omalizumab | 5.014 | 0.0108 | Riviere et al., (2011) | 0.62 | Prescribing Info | 150 ^c^ | Riviere et al., (2011) |
| Omalizumab | 5.014 | 0.0180 | Riviere et al., (2011) | 0.62 | Prescribing Info | 300 ^c^ | Riviere et al., (2011) |

^a^ Dose in units of mg/m^2^ converted from units of IU/m^2^ (0.343 IU/pg; Kirchner et al., 1998).

^b^ Dose in units of mg converted from units of IU (4 x 10^6^ IU/mg; Konrad et al., 1990).

^c^ Dose in units of mg.

^d^ Dose in units of mIU.

^e^ Dose converted from units of IU/kg (0.33 IU/mg; Zeisel et al., 1992).

^f^ Dose in units of mIU/kg.

^g^ Dose in units of mg/m^2^ converted from units of IU/m^2^ (0.33 IU/mg; Zeisel et al., 1992).

^h^ Dose converted from units of IU/kg (160000 IU/mg).

^i^ Dose in units of % dose.

^j^ Weighted mean value from reported intravenous clearance values from Konrad et al., (1992) and Piscitelli et al., (1996).

^k^ Weighted mean value from reported intravenous clearance values from Radwanski et al., (1998); Huhn et al., (1996); Andersen et al., (1999) and Fuchs et al., (1996).

^l^ Value fitted using Parameter Estimation facility and Full PBPK model for Other Proteins in the Simcyp Simulator V13R2.

B

A


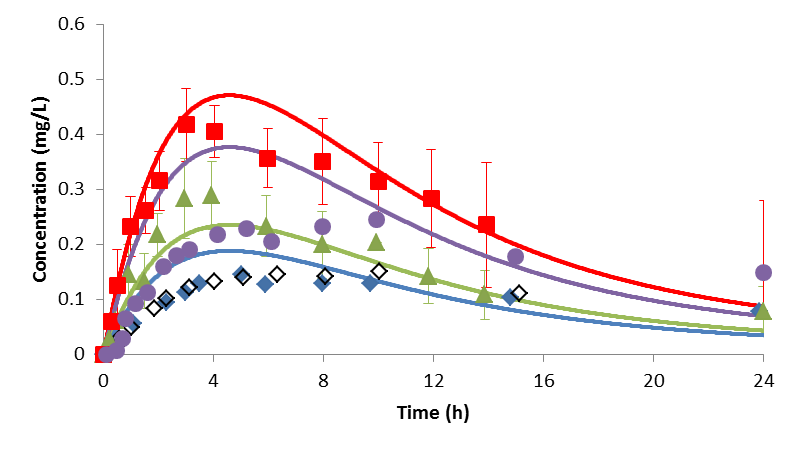

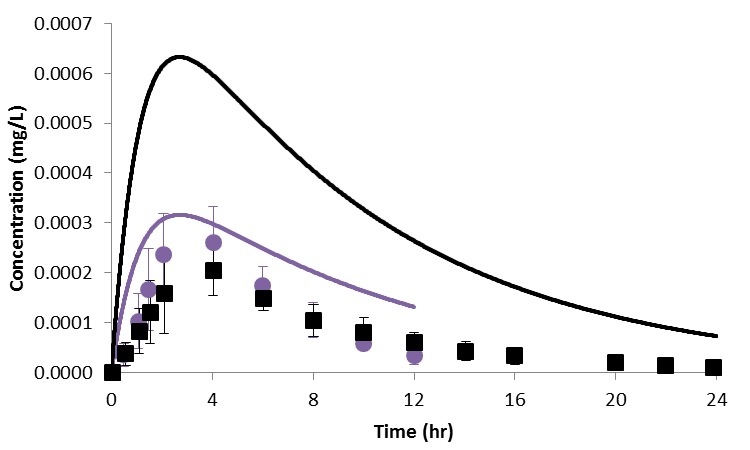

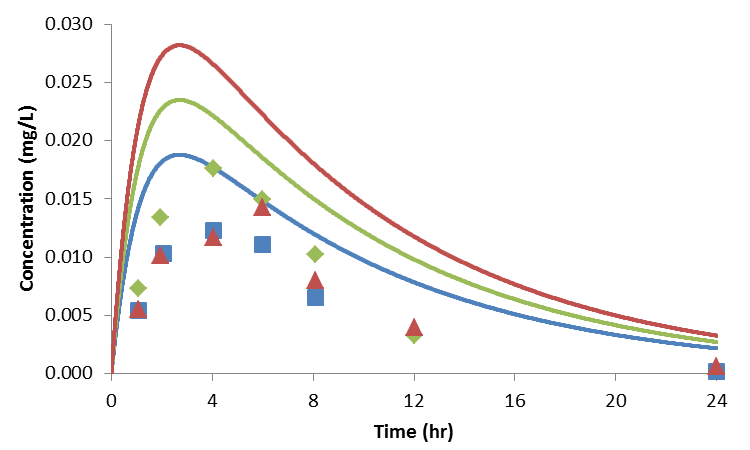

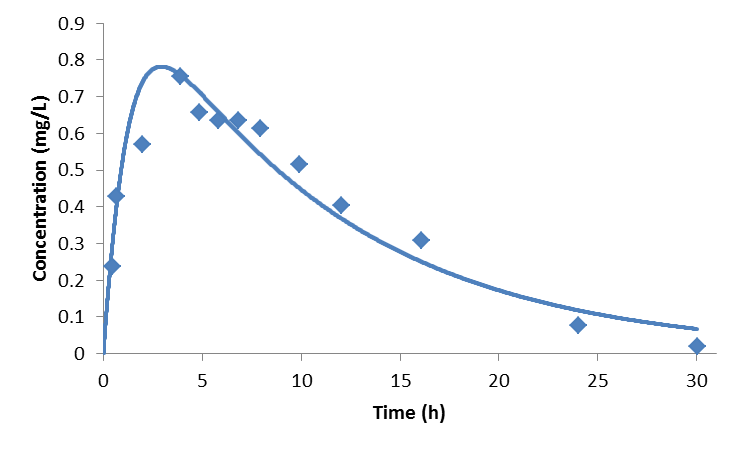

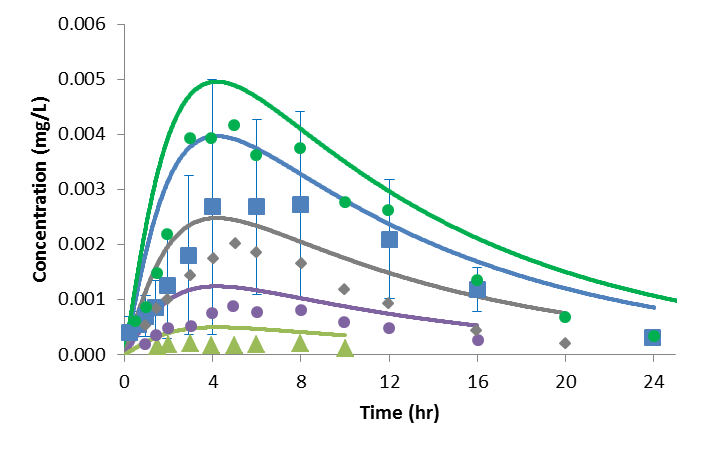

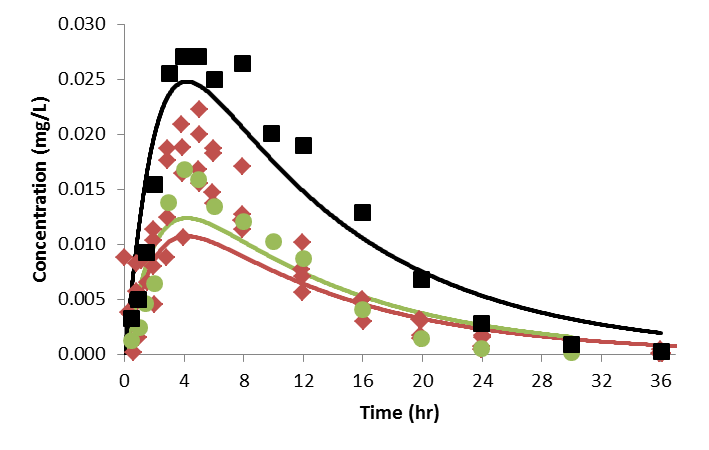

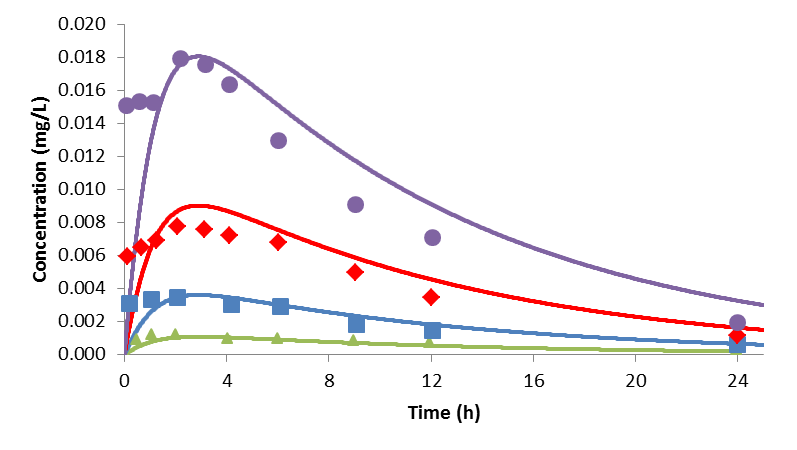

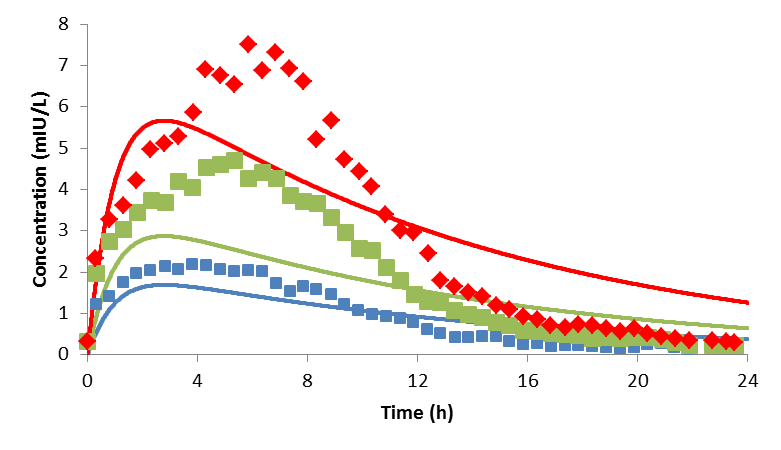

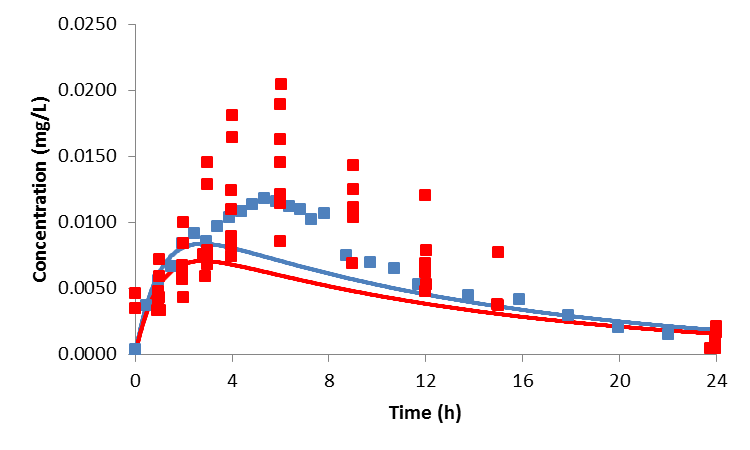

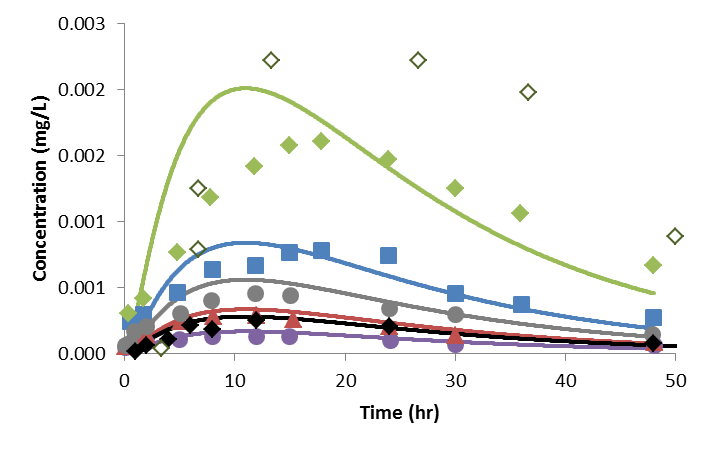

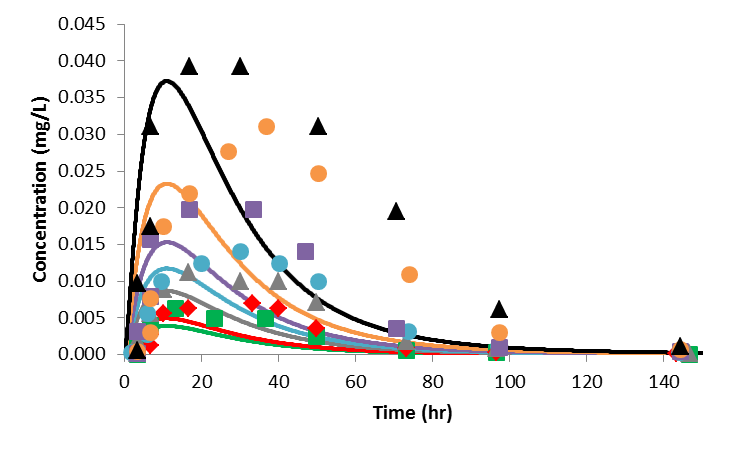

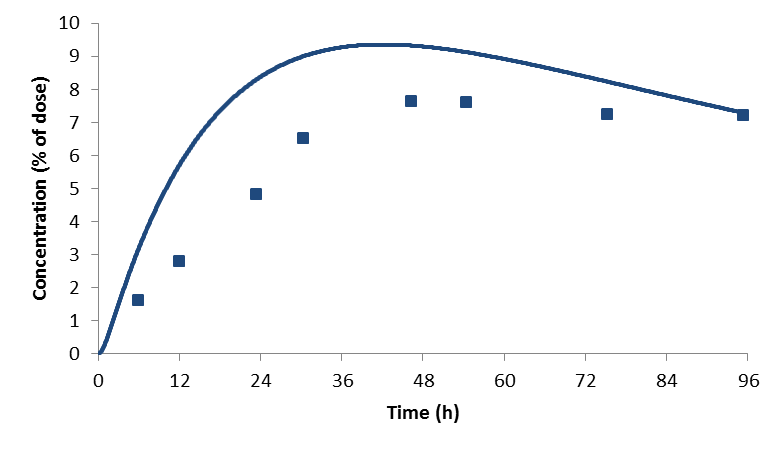

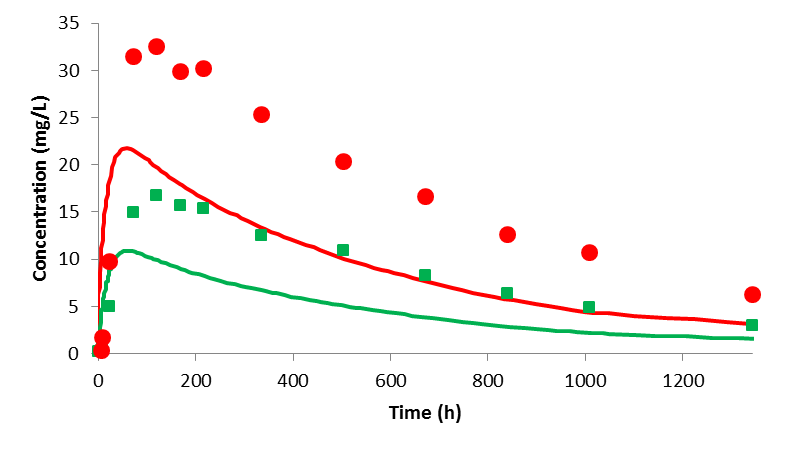

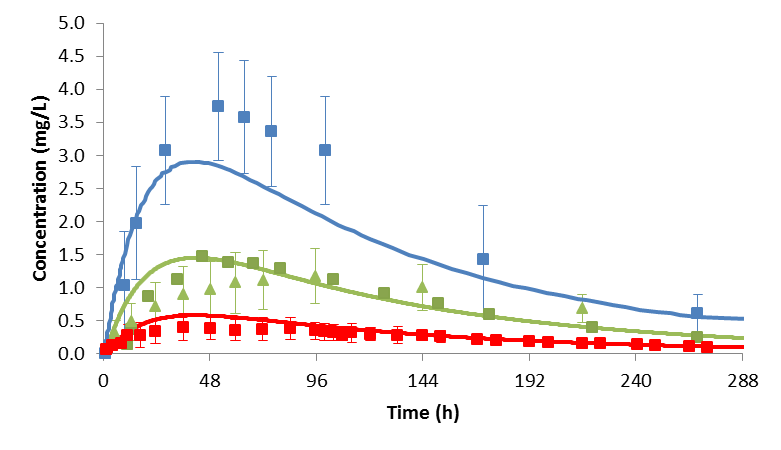

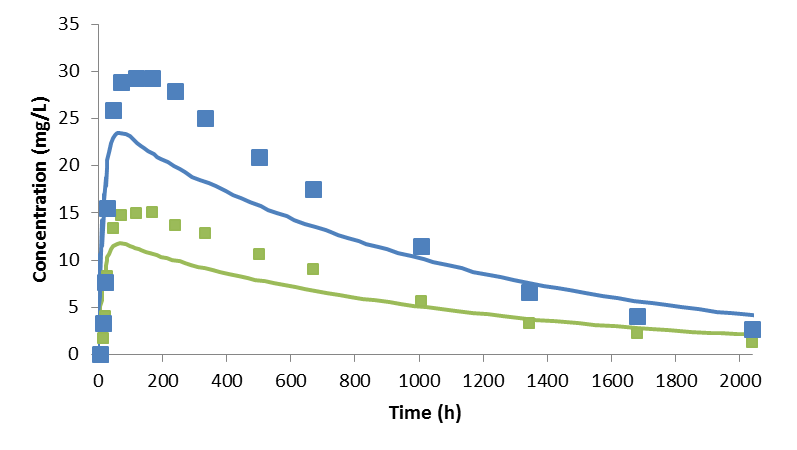


▬ 600 mIU
▬ 1200 mIU ▬ 1800 mIU

▬ 3 µg/kg
▬ 10 µg/kg ▬ 25 µg/kg ▬ 50 µg/kg

▬ 3 mg
▬ 3.75 mg ▬ 4.5 mg

▬ 1.75 mg
▬ 25 µg/kg ▬ 50 µg/kg

▬ 1 µg/kg ▬ 2.5 µg/kg ▬ 5 µg/kg ▬ 8 µg/kg ▬ 10 µg/kg

▬ 100 mg

▬ 0.03 mg/m^2^ ▬ 0.06 mg/m^2^

**hGH**

**IL-11**

**IL-10**

**IL-10**

**Anakinra**

**IL-2**

**IL-2**

▬ 40 µg/kg ▬ 50 µg/kg ▬ 80 µg/kg ▬ 100 µg/kg

**IGF-1**

H

G

F

E

C

D

**Supplemental Figure 1: Predicted and observed plasma concentrations for TPs following SC dosing.** A: IGF-1; B & C: IL-2; D: Anakinra: E & F: IL-10; G: IL-11; H & I: hGH; J & K: EPO; L: Albumin; M; Tralokinumab; N: Etanercept; O: Omalizumab. Symbols represent observed data; lines represent predicted data. A: blue, open diamond, green, purple and red symbols/lines = 40, 40, 50, 80 and 100 μg/kg doses (Grahnen et al., 1993; Wilton et al., 1991; Fouque et al., 1995); B: black and purple symbols/lines = 0.03 and 0.06 mg/m^2^ doses (Kirschner et al., 1998); C: blue, green and red symbols/lines = 3, 3.75 and 4.5 mg doses (Piscitelli et al., 1996); D: Yang et al., 2003; E: olive green, purple, grey, blue and green symbols/lines = 1, 2.5, 5, 8 and 10 μg/kg doses (Huhn et al., 1997; Chakraborty et al., 1999); F: red, green and black symbols/lines = 1.75 mg, 25 and 50 µg/kg doses (Radwanski et al., 1998; Huhn et al., 1997); G: green, blue, red and purple symbols/lines = 3, 10, 25 and 50 μg/kg doses (Aoyama et al., 1997); H: blue, green and red symbols/lines = 600, 1200 and 1800 mIU doses (Janssen et al., 1999); I: red and blue symbols/lines = 1.3 mg/m^2^ and 0.033 mg/kg doses (Zeisel et al., 1992; Laursen et al., 1996); J: purple, black, red, grey, blue, green and open diamond symbols/lines = 0.188, 0.313, 0.375, 0.625, 0.938, 1.88 and 1.88 μg/kg doses (Sans et al., 2000; Salmonson et al., 1999; McMahon et al., 1990; Ramakrishnan et al., 2004); K: green, red, grey, blue, purple, orange and black symbols/lines = 2.81, 3.75, 5.63, 7.50, 8.44, 11.3 and 15 μg/kg doses (Ramakrishnan et al., 2004); L: Hollander et al., 1961; M: green and red symbols/lines = 150 and 300 mg doses (Oh et al., 2010); N: red, green and blue symbols/lines = 10, 25 and 50 mg doses (Yi et al., 2012; Sullivan et al., 2006; Korth-Bradley et al., 2000; Zhou et al., 2005); O: green and blue symbols/lines = 150 and 300 mg doses (Riviere et al., 2011).

▬ 10 mg
▬ 25 mg ▬ 50 mg

▬ 150 mg ▬ 300 mg

▬ 150 mg ▬ 300 mg

▬ 100 %dose

**Albumin**

▬ 0.188 µg/kg ▬ 0.313 µg/kg ▬ 0.375 µg/kg ▬ 0.625 µg/kg ▬ 0.938 µg/kg ▬ 1.88 µg/kg

▬ 2.81 µg/kg ▬ 3.75 µg/kg ▬ 5.63 µg/kg ▬ 7.50 µg/kg ▬ 8.44 µg/kg ▬ 11.3 µg/kg ▬ 15.0 µg/kg

▬ 1.3 mg/m^2^ ▬ 0.033 mg/kg

**Omalizumab**

**Etanercept**

**Tralokinumab**

**EPO**

**EPO**

**hGH**

O

N

M

L

K

I

J


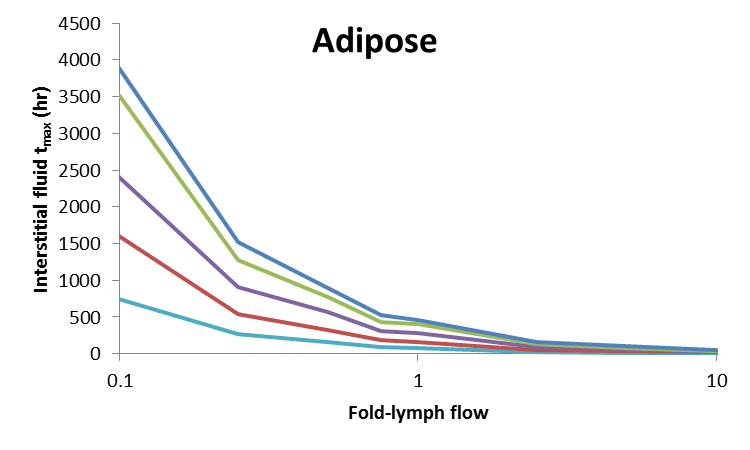

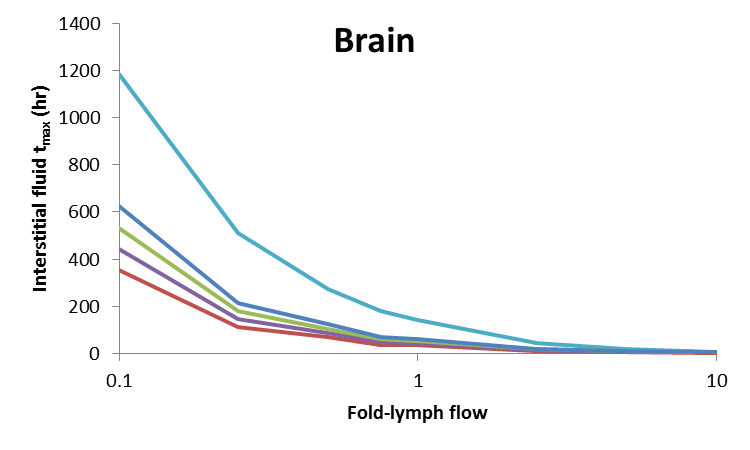

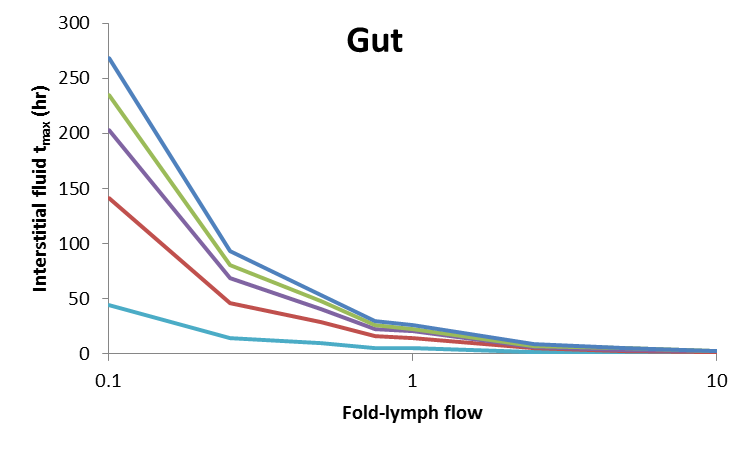

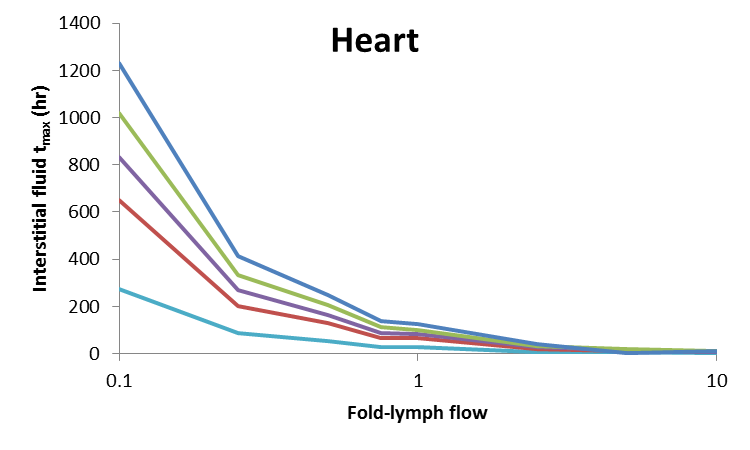

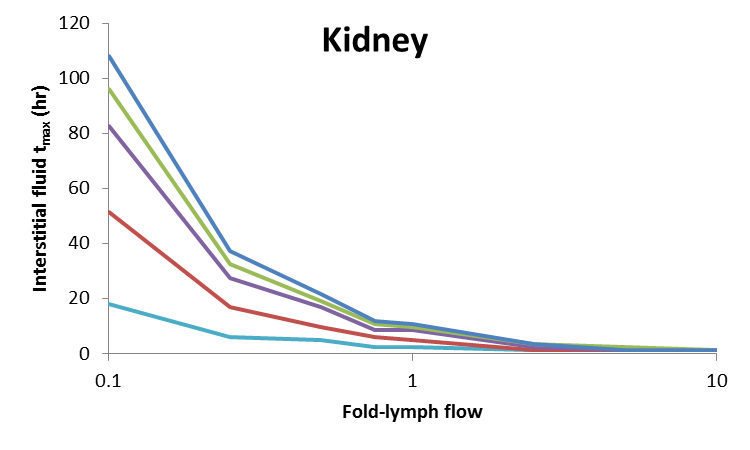

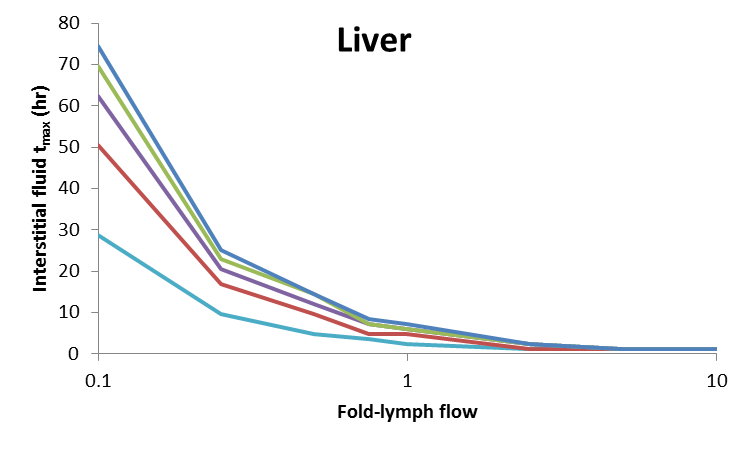

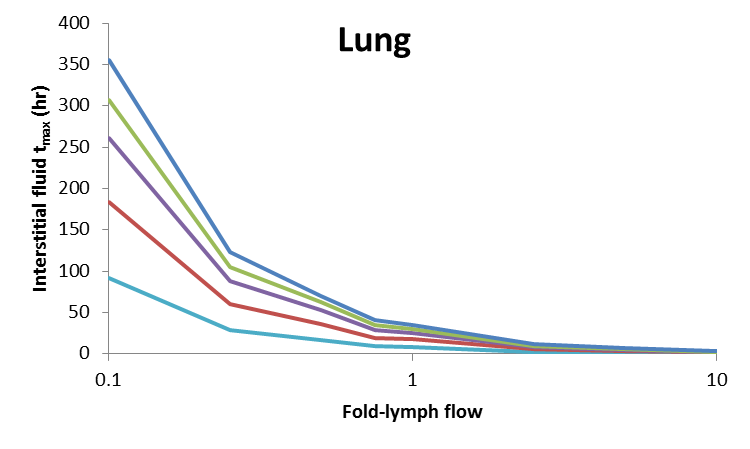

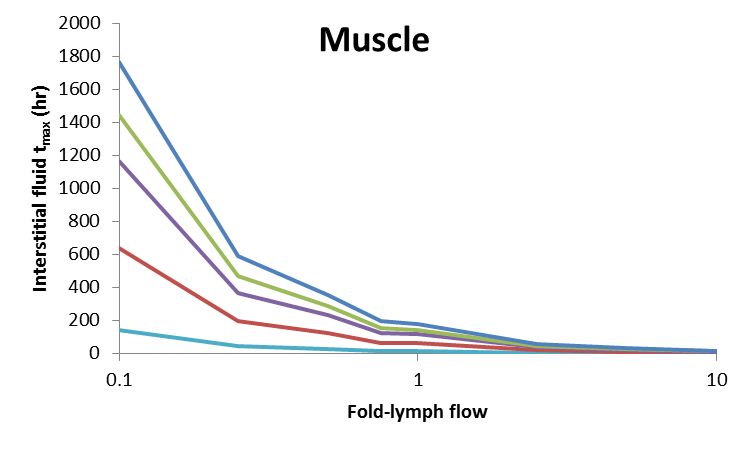

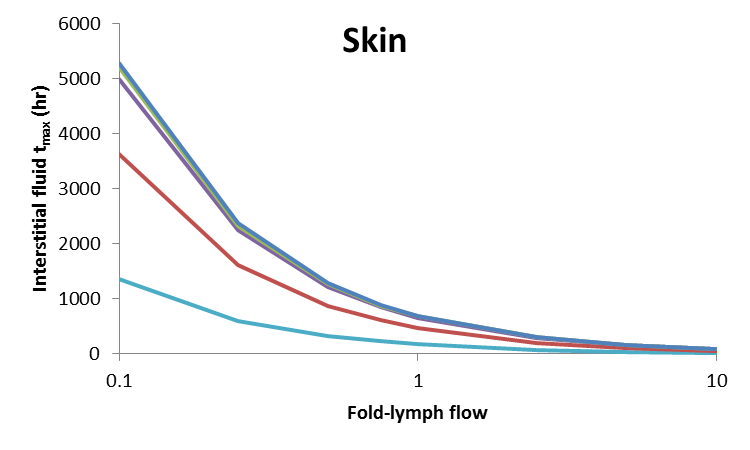

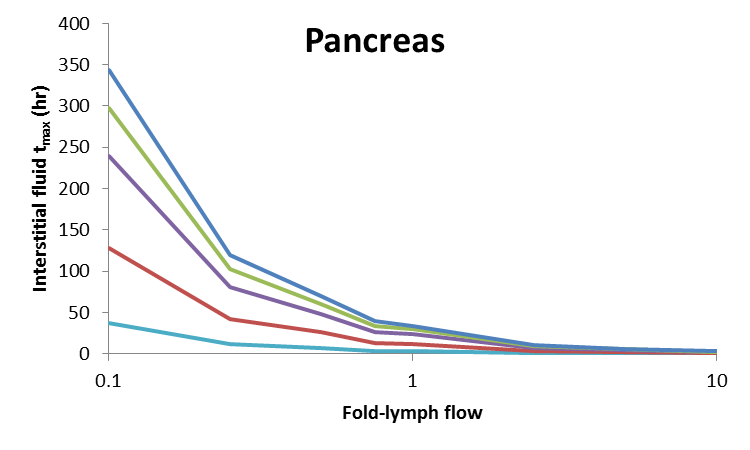


**Supplemental Figure 2: Sensitivity analysis: Impact of lymph flow on tissue interstitial fluid t_max_ for a range of theoretical proteins.** Lymph flow was manually altered by 0.1, 0.25, 0.5, 0.75, 1, 2.5, 5 and 10-fold and t_max_ in tissue interstitial fluid for theoretical proteins with a range of size. Light blue, red, purple, green and dark blue lines represent theoretical proteins with 1, 2, 3, 4 and 5 nm hydrodynamic radius, respectively.


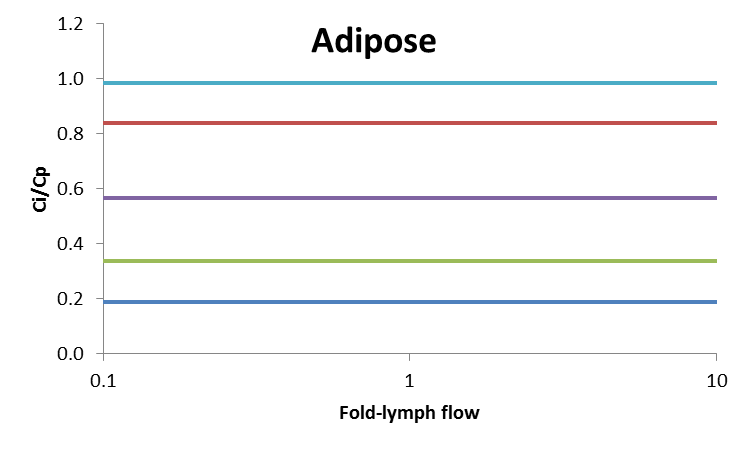

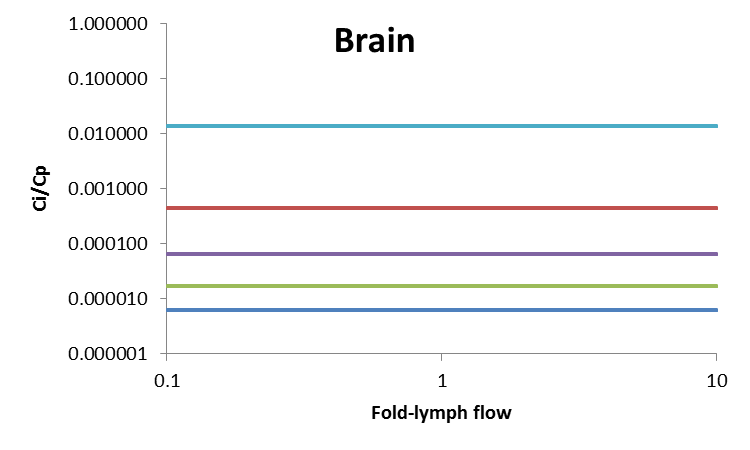

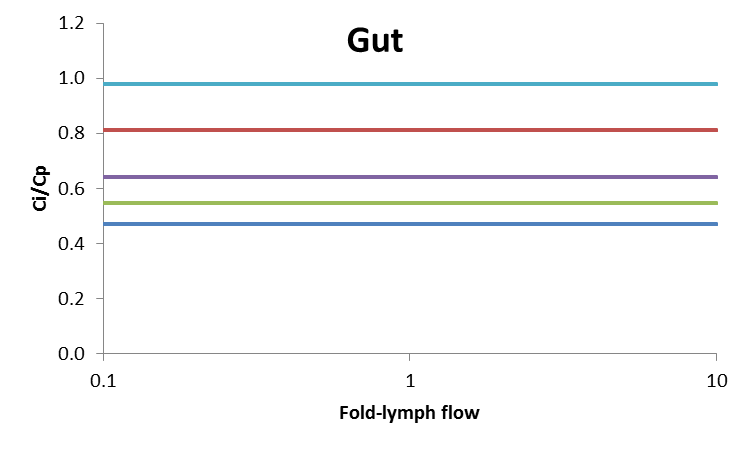

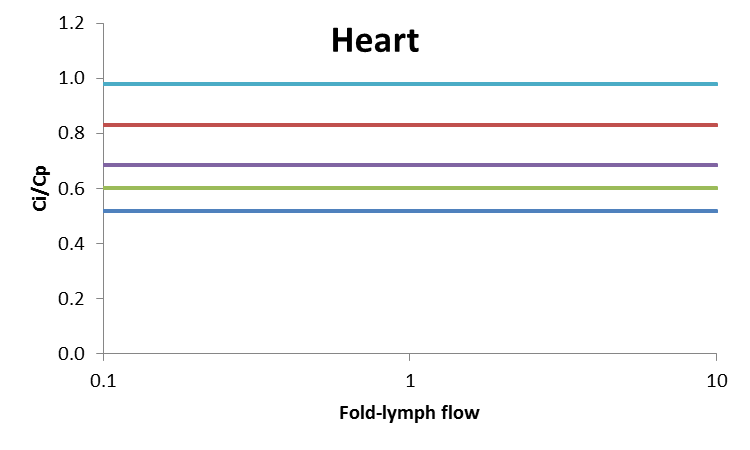

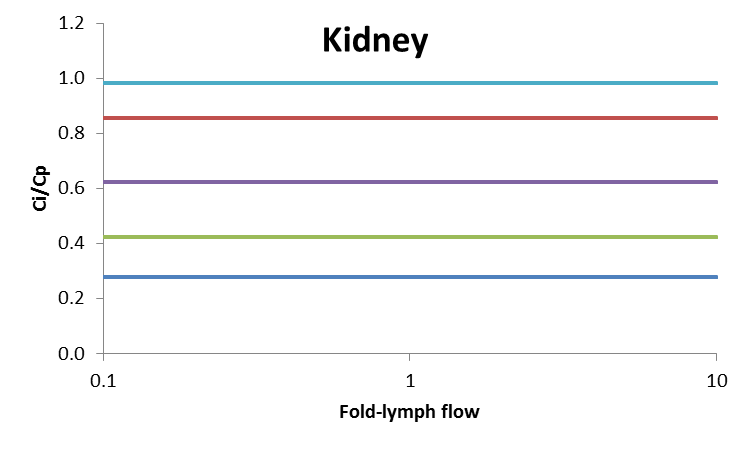

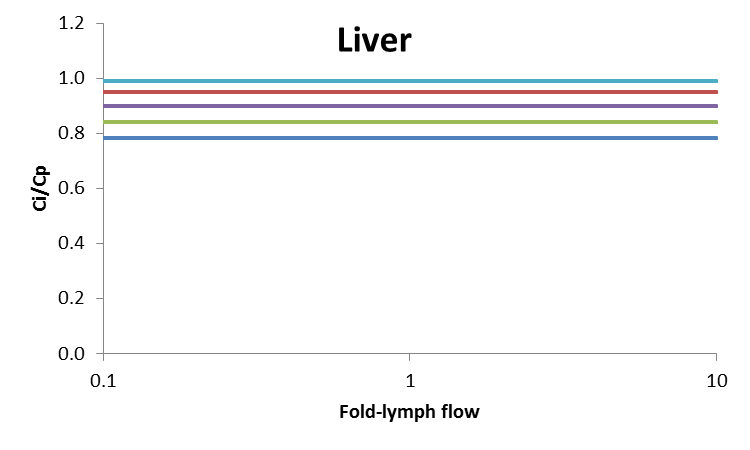

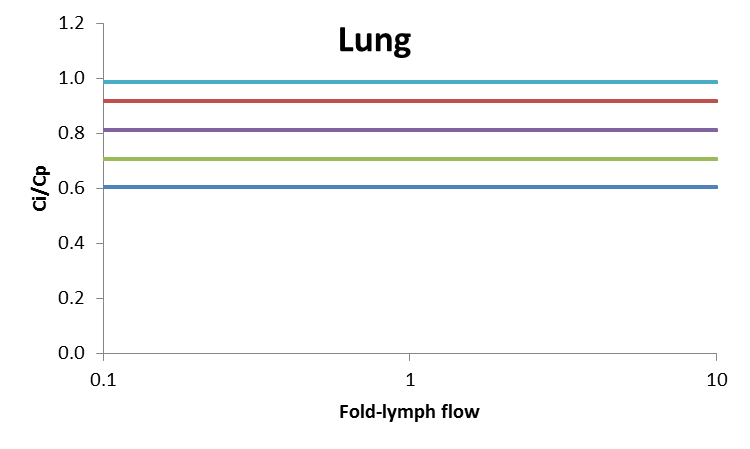

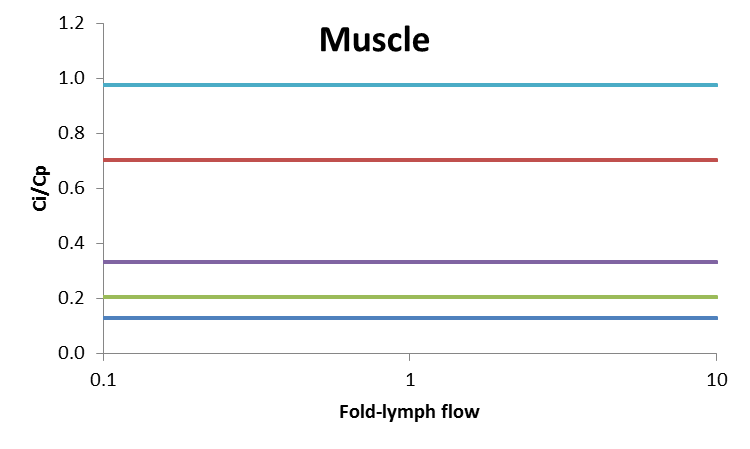

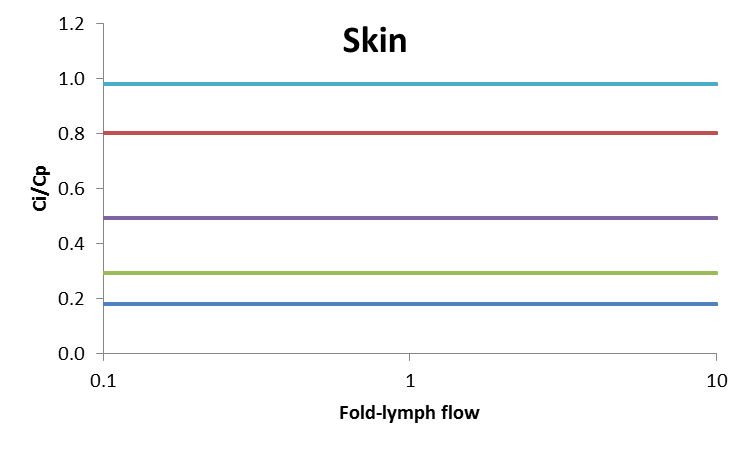

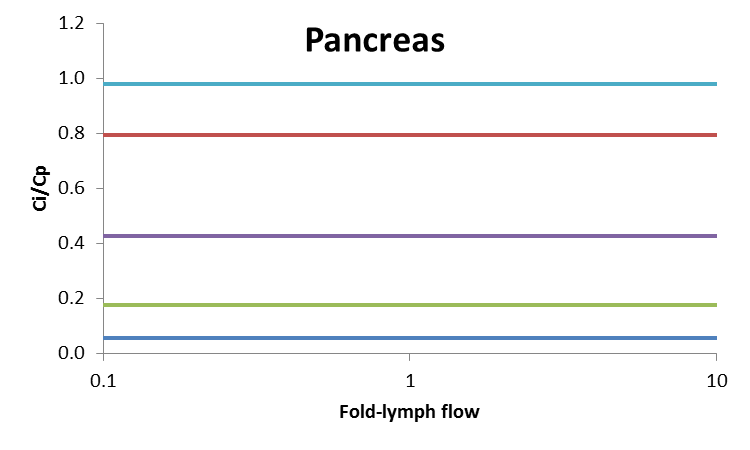


**Supplemental Figure 3: Sensitivity analysis: Impact of lymph flow on tissue Ci/Cp for a range of theoretical proteins.** Lymph flow was manually altered by 0.1, 0.25, 0.5, 0.75, 1, 2.5, 5 and 10-fold and tissue Ci/Cp for theoretical proteins with a range of size. Light blue, red, purple, green and dark blue lines represent theoretical proteins with 1, 2, 3, 4 and 5 nm hydrodynamic radius, respectively.
